# N6-methyladenosine dynamics in neurodevelopment and aging, and its potential role in Alzheimer’s disease

**DOI:** 10.1186/s13059-020-02249-z

**Published:** 2021-01-05

**Authors:** Andrew M. Shafik, Feiran Zhang, Zhenxing Guo, Qing Dai, Kinga Pajdzik, Yangping Li, Yunhee Kang, Bing Yao, Hao Wu, Chuan He, Emily G. Allen, Ranhui Duan, Peng Jin

**Affiliations:** 1 Department of Human Genetics, School of Medicine, Emory University, Atlanta, GA 30322 USA; 2 Department of Biostatistics and Bioinformatics, School of Public Health, Emory University, Atlanta, GA 30322 USA; 3 Department of Chemistry, University of Chicago, Chicago, IL 60637 USA; 4 Center for Medical Genetics, School of Life Sciences, Central South University, Changsha, 410078 Hunan China

**Keywords:** Epitranscriptomics, m^6^A, Neurodevelopment, Aging, Alzheimer’s, Regulation of mRNA levels, Regulation of protein levels, Alternative 3′UTR

## Abstract

**Background:**

N6-methyladenosine (m^6^A) modification is known to impact many aspects of RNA metabolism, including mRNA stability and translation, and is highly prevalent in the brain.

**Results:**

We show that m^6^A modification displays temporal and spatial dynamics during neurodevelopment and aging. Genes that are temporally differentially methylated are more prone to have mRNA expression changes and affect many pathways associated with nervous system development. Furthermore, m^6^A shows a distinct tissue-specific methylation profile, which is most pronounced in the hypothalamus. Tissue-specific methylation is associated with an increase in mRNA expression and is associated with tissue-specific developmental processes. During the aging process, we observe significantly more m^6^A sites as age increases, in both mouse and human. We show a high level of overlap between mouse and human; however, humans at both young and old ages consistently show more m^6^A sites compared to mice. Differential m^6^A sites are found to be enriched in alternative untranslated regions of genes that affect aging-related pathways. These m^6^A sites are associated with a strong negative effect on mRNA expression. We also show that many Alzheimer-related transcripts exhibit decreased m^6^A methylation in a mouse model of Alzheimer’s disease, which is correlated with reduced protein levels.

**Conclusions:**

Our results suggest that m^6^A exerts a critical function in both early and late brain development in a spatio-temporal fashion. Furthermore, m^6^A controls protein levels of key genes involved in Alzheimer’s disease-associated pathways, suggesting that m^6^A plays an important role in aging and neurodegenerative disease.

## Background

N6-methyladenosine (m^6^A) in mRNA is a reversible modification and can mediate its function in a dynamic manner [1, 2]. m^6^A has been shown to affect RNA metabolism including RNA degradation [3], translation [4–6], RNA splicing [7], and nuclear export [8, 9]. m^6^A is installed by methyltransferases (writers), removed by demethylases (erasers), and recognized by m^6^A binding proteins (readers). Currently, known writers include methyltransferase-like protein 3 (METTL3), METTL14, and Wilms tumor 1-associating protein [10, 11], while erasers include AlkB homolog 5 [12] and obesity-associated protein FTO [13], and readers include YTHDF1 [5], YTHDF2 [14], YTHDF3 [15, 16], YTHDC1 [17], YTHDC2 [18], hnRNP A2/B1 [19], hnRNPC [20], and hnRNPG [21].

m^6^A is an abundant RNA modification in the brain, and studies have implicated m^6^A in neurogenesis [22], learning and memory [23], brain development [24–27], and axon regeneration [28]. Importantly, many players in the m^6^A pathway (e.g., hnRNP A2/B1, METTL14, YTHDF 1,2,3) have been implicated as critical factors in neuronal function, and tight regulation of brain processes by m^6^A are required for proper brain development [29]. Furthermore, various m^6^A players have been found to be mutated or dysregulated both in human neurological disorders, such as epilepsy, intellectual disability, depression, and schizophrenia, and in neurodevelopmental disorders [30–32]. However, the precise underlying mechanisms are yet to be fully established. For example, m^6^A may likely play a pivotal role in Alzheimer’s disease (AD), as it is an age-related neurodegenerative disease and is defined by changes in synapses where m^6^A is known to exert its regulatory function. However, the role of m^6^A in the brain still requires further elucidation.

Both epigenetic and transcriptional mechanisms have been shown to impact brain development. For example, epigenetic mechanisms, including DNA methylation and histone modification, are crucial in neurodevelopmental reprogramming [33, 34], and epigenetic disruption results in neurodevelopmental disorders [35]. Also, in response to changes in neuronal function and activity, maintaining proper mRNA levels through degradation and stabilization is critical for proper brain function. Disruptions in RNA metabolism, including mRNA splicing, are associated with aging and age-related disorders, such as frontotemporal lobar dementia (FTD) [36], Parkinson’s disease [37], and Alzheimer’s disease [38]. Indeed, in addition to epigenetic and transcriptional control, epitranscriptomic regulation could potentially provide yet another layer of regulation.

Here we investigated the dynamic regulation of m^6^A in the developing and aging mouse and human brains. This study was further extended by determining the landscape and function of m^6^A in the context of Alzheimer’s disease. Altogether, by employing next-generation sequencing, bioinformatic and genetic tools, this study reveals RNA m^6^A methylation plays a pertinent role in the regulation of the aging mammalian brain by regulating mRNA expression levels of transcripts involved in aging through the 3′ untranslated region (UTR), while also playing a key role in the occurrence of Alzheimer’s disease by regulating protein levels of AD-associated transcripts.

## Results

### Profiling m^6^A dynamics during neurodevelopment and aging in mouse

**Fig. 1:**
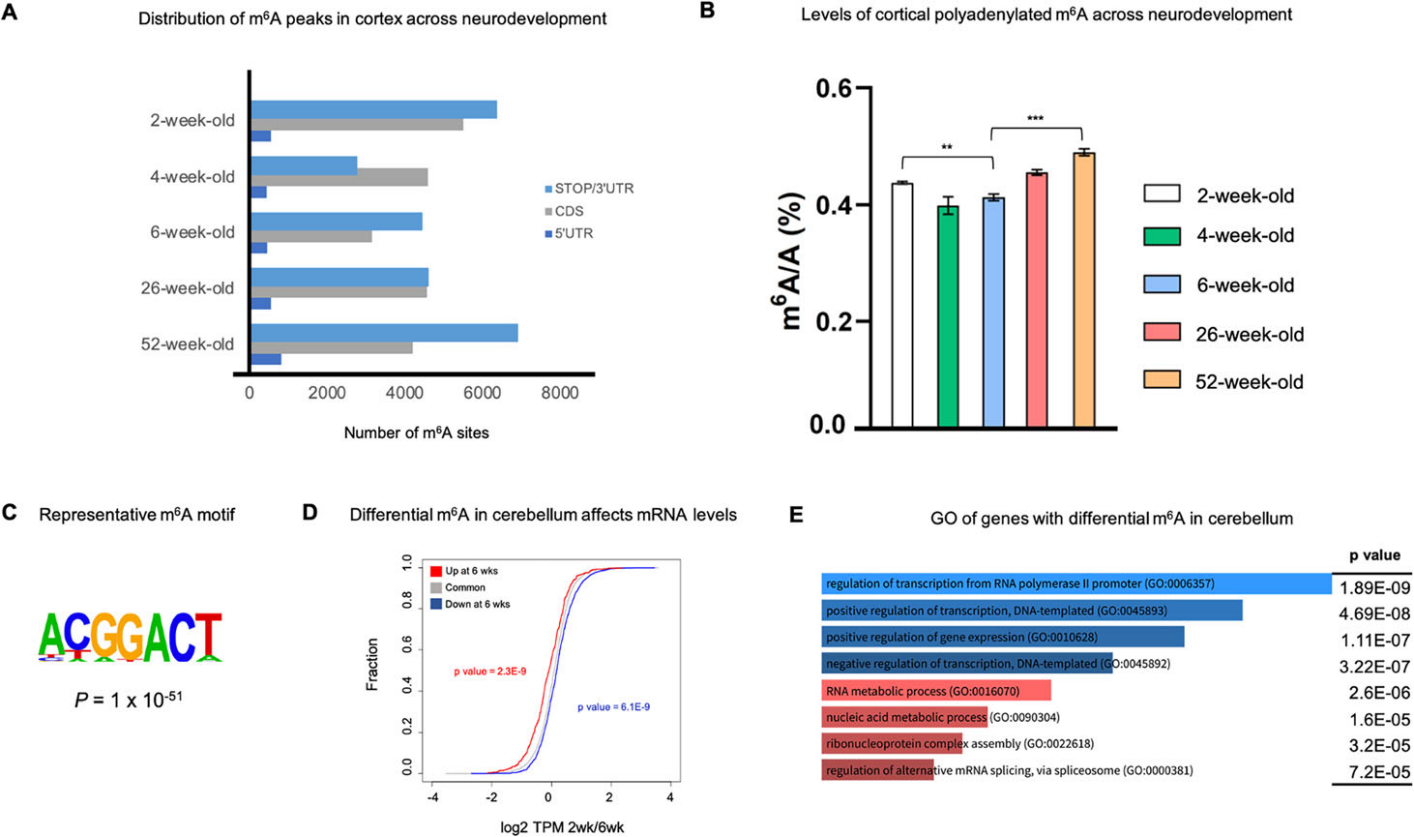
Characterizing m^6^A in early to late brain development. **a** Number of cortical m^6^A sites in each gene region across 2-week, 4-week, 6-week, 26-week, and 52-week-old mice. **b** LC-MS/MS quantification of m^6^A levels in poly (A) + mRNA across neurodevelopment. **c** Motif identified using the 2-week cortex but is representative of all tissues and time points. **d** Cumulative frequency plot showing how methylation status correlates with mRNA expression levels. Genes that are hypermethylated at 6 weeks have a smaller 2wk/6wk log2 TPM ratio. **e** Gene ontology analysis of enriched biological processes of genes that are hypomethylated (blue) or hypermethylated (red) at 6 weeks compared to 2 weeks

To determine the landscape of m^6^A in early postnatal brain development and aging, we profiled the m^6^A transcriptome using m^6^A-seq in 4 different brain regions (cerebral cortex, cerebellum, hypothalamus, and hippocampus) across five different developmental time points (2-week, 4-week, 6-week, 26-week, and 52-week-old B6 mice) (Additional file 2: Table S1 shows the number of uniquely mapped reads per sample). Poly (A) + RNA was isolated to generate both RNA-seq and m^6^A-seq datasets. 11,793, 8179, 7089, 8269, and 12,080 high confidence peaks were detected in the cortex at 2-week, 4-week, 6-week, 26-week, and 52-week-old B6 mice, respectively (Additional file 3: Table S2 for the number of m^6^A peaks for each time point and brain region and Additional file 1: Fig. S1). PCA plots confirmed the reproducibility between replicates and differences amongst samples (Additional file 1: Fig. S2). We detected the majority of m^6^A marks in the coding region, and the stop codon/3′ untranslated region (UTR) (Fig. 1a). Furthermore, across all four tissues, we observed m^6^A peaks decreasing from 2 to 6 weeks, after which m^6^A peaks increased at 26 weeks and continued at 52 weeks. This trend was further confirmed by quantifying m^6^A% ratio using LC-MS/MS (Fig. 1b). Lastly, we performed a de novo motif search on the detected m^6^A sites and found them to be enriched in the consensus GGAC m^6^A motif that was reported previously, confirming the quality of our data (Fig. 1c) [25, 39].

### Temporal dynamics of m^6^A methylation during neurodevelopment

To determine the temporal distribution of m^6^A methylation during neurodevelopment, we focused the m^6^A methylome in the 2-week and 6-week-old mice. We performed the comparison of m^6^A profiles in the cerebellum, cortex, hippocampus, and hypothalamus and consistently observed a significant decrease in m^6^A methylation at 6 weeks compared to 2 weeks, with the most significant change being observed in the cerebellum. We detected 410 and 1557 transcripts that have differentially increased or decreased methylation at 6 weeks compared to 2 weeks, respectively, in the cerebellum, *p* value = 6.1E−9 (Additional file 4: Table S3 shows differential sites throughout neurodevelopment in all four tissues). We also observed a correlation between m^6^A and mRNA levels. Differentially methylated transcripts have significantly different mRNA expression compared to transcripts that are methylated at both time points. That is, transcripts that are hypomethylated at 6 weeks tend to have significantly lower mRNA expression levels than transcripts that experience no change in methylation from 2 to 6 weeks; the reverse is true for transcripts that become hypermethylated at 6 weeks (Fig. 1d). This suggests that m^6^A exerts a stronger effect on the steady level of mRNA in these differentially methylated transcripts than in the transcripts that are methylated at both time points. Overall, hypermethylated transcripts tend to be involved in RNA metabolic processes, whereas hypomethylated transcripts are associated with regulation of gene expression (Fig. 1e), suggesting that the dynamic nature of m^6^A is critical in different pathways to ensure proper early postnatal brain development.

Also, given that m^6^A has a role in mRNA alternative splicing, we investigated whether there is an association between m^6^A and splicing across neurodevelopment. We performed deep RNA-seq across neurodevelopment and find that a subset of exonic m^6^A sites are associated with exon inclusion events. We find a similar number of these events across neurodevelopment and those events occur in genes are primarily involved in synaptogenesis. We also note that these events occur in three m^6^A-binding proteins (YTHDF1, 2, 3) (Additional file 5: Table S4 for a list of m^6^A-associated exon inclusion events and Additional file 1: Fig. S3 for screenshots from the UCSC genome browser).

Consistent with previous findings, our analyses suggest that m^6^A is able to exert its function in multiple ways, and we show that m^6^A is associated with both mRNA expression and mRNA splicing across neurodevelopment. The dynamic nature of m^6^A is critical in regulating mRNA expression and seems to be a major role of m^6^A in neurodevelopment, whereas m^6^A-associated exon inclusion events are fewer and tend to be continuous across neurodevelopment. Thus, m^6^A seems to have a greater impact on mRNA levels than on splicing.

### Spatial dynamics of m^6^A methylation during neurodevelopment

**Fig. 2:**
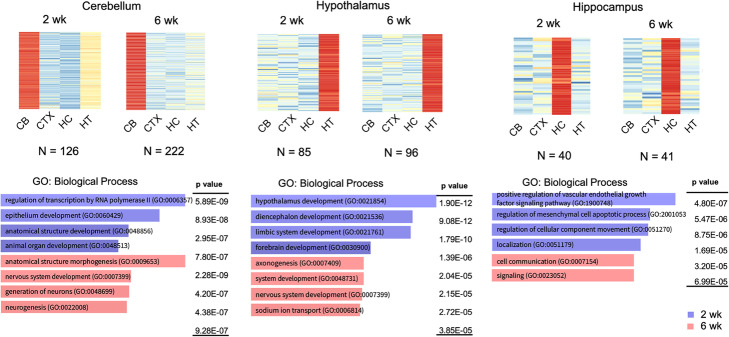
Region-specific m^6^A methylation in the brain. Heatmaps correlating mRNA expression levels with the presence of methylation. Cerebellum-, hypothalamus-, and hippocampus-specific methylation correlates with the highest mRNA level in that tissue compared to the others. *N* refers to the number of specific methylation events for a specific tissue at a specific developmental time point. Gene ontology analysis of enriched biological processes of genes that are specifically methylated at 2 weeks (blue) or at 6 weeks (red)

Next, to understand the spatial effect of m^6^A methylation in the mouse brain across development, we profiled the modification across 4 brain tissues—cerebellum, hypothalamus, hippocampus, and cortex. At both 2 weeks and 6 weeks, we observed a strong propensity for tissue-specific methylation in the cerebellum, hypothalamus, and hippocampus but not in the cortex. At 2 weeks, we find 126, 85, and 40 transcripts harboring tissue-specific m^6^A sites in the cerebellum, hypothalamus, and hippocampus, respectively. Similar tissue-specific methylation was also detected at 6 weeks, with 222, 96, and 41 uniquely methylated transcripts in the cerebellum, hypothalamus, and hippocampus, respectively (evidence of tissue-specific methylation is shown in Additional file 1: Fig. S4 coverage tracks). Interestingly, at both 2 and 6 weeks, we observed that tissue-specific m^6^A methylation correlates with an increase in mRNA expression in that tissue relative to the others (Fig. 2, top panel) (Additional file 6: Table S5 shows tissue-specific m^6^A methylation and mRNA levels). For cerebellum-specific methylation at 2 weeks, we noted that a significant proportion of these transcripts are involved in the development and transcriptional regulation pathways. More strikingly, hypothalamus-specific methylation occurs in genes involved in hypothalamus development. Hippocampus-specific methylation was detected in 3 out of 6 genes involved in the positive regulation of vascular endothelial growth factor signaling pathway. At 6 weeks, in the cerebellum, tissue-specific methylation is present in genes associated with nervous system development and neurogenesis. In the hypothalamus, significant gene ontologies are associated with system development and metal ion transport, whereas cell communication and signaling terms are important in the hippocampus (Fig. 2, bottom panel). These results suggest that m^6^A differentially marks mRNAs to ensure brain tissue-specific expression at particular times across development.

### m^6^A methylation dynamics during brain aging

**Fig. 3:**
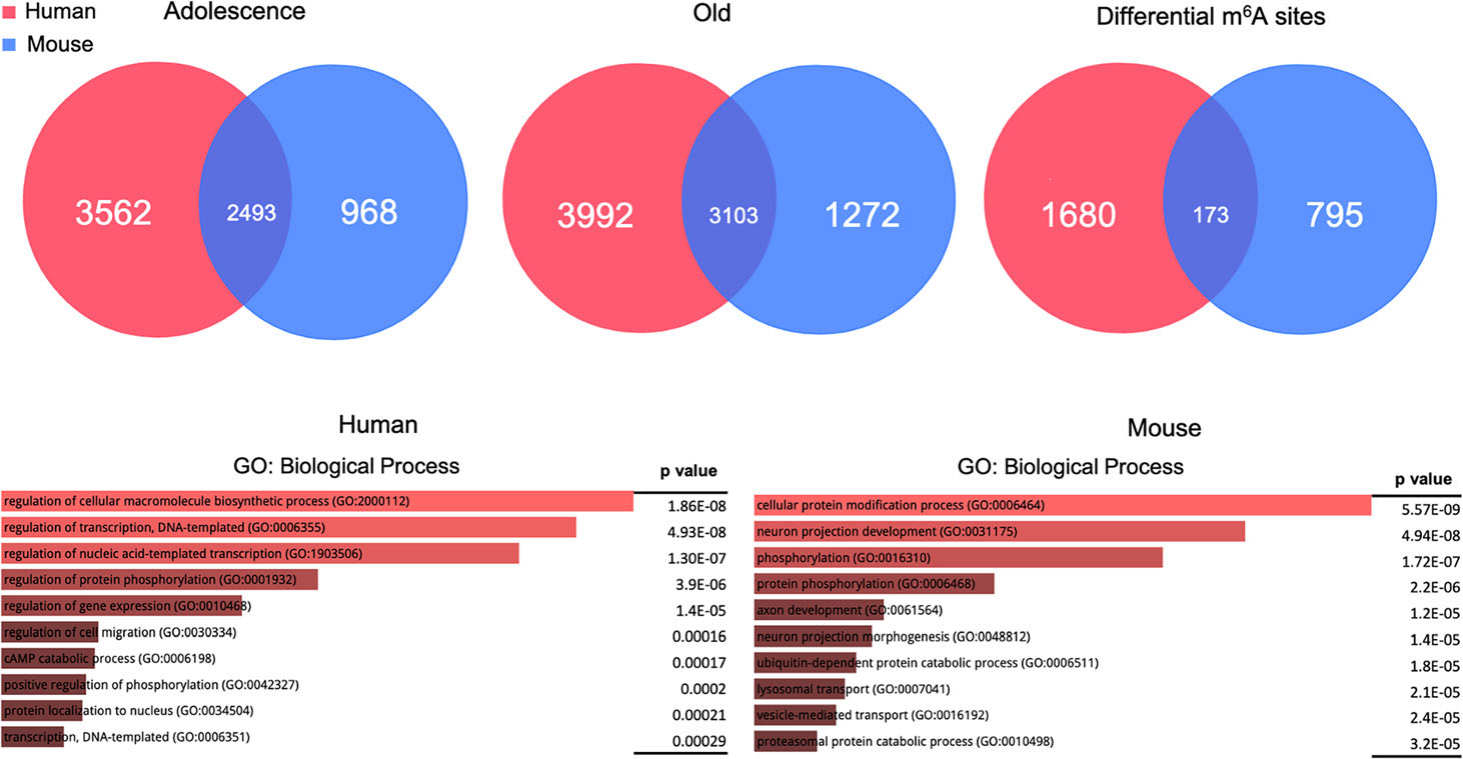
Comparison of human and mouse genes containing 3′ UTR m^6^A in adolescent and aged brains. Top panel: Venn diagrams showing the number of methylated genes at each developmental time point in human (red) and in mouse (blue). Differential sites are those that are hypermethylated in old vs adolescence. Bottom panel: Gene ontology analysis of enriched biological processes of genes that are methylated in human and mouse

Given the role of m^6^A in neurodevelopment, we next investigated the m^6^A dynamics during aging. To this end, we compared the m^6^A profiles of 6-week to 52-week-old mice cerebral cortex and also profiled the m^6^A transcriptome in both adolescent and old postmortem human brain tissue region BA9. In both comparisons, we observed a significant increase in m^6^A methylation from adolescent to old, and no statistically significant hypomethylated transcripts were identified in the old samples. In human, we observed 6055 genes that were marked with m^6^A in the adolescent sample which increased to 7095 in old. Interestingly, we identified significantly less m^6^A-marked genes in mouse. Overall, we detected m^6^A methylation in 3461 genes at 6 weeks compared with 4375 genes at 52 weeks old. Also, we identified a large number of genes that are methylated in both human and mouse, suggesting a high degree of conservation and an important role for m^6^A in the aging process. Furthermore, in humans, we identified about 1800 transcripts with hypermethylation at old compared to young. In mice, we see the same trend; however, only about 960 differential peaks were identified (Fig. 3, top panel) (Additional file 7: Table S6 shows differentially methylated transcripts in mouse and human). A gene ontology analysis revealed that these differentially marked genes are involved in processes such as regulation of protein modification processes, transcription, and neuron projection (Fig. 3, bottom panel).

### m^6^A methylation and differential UTR usage during brain aging

**Fig. 4:**
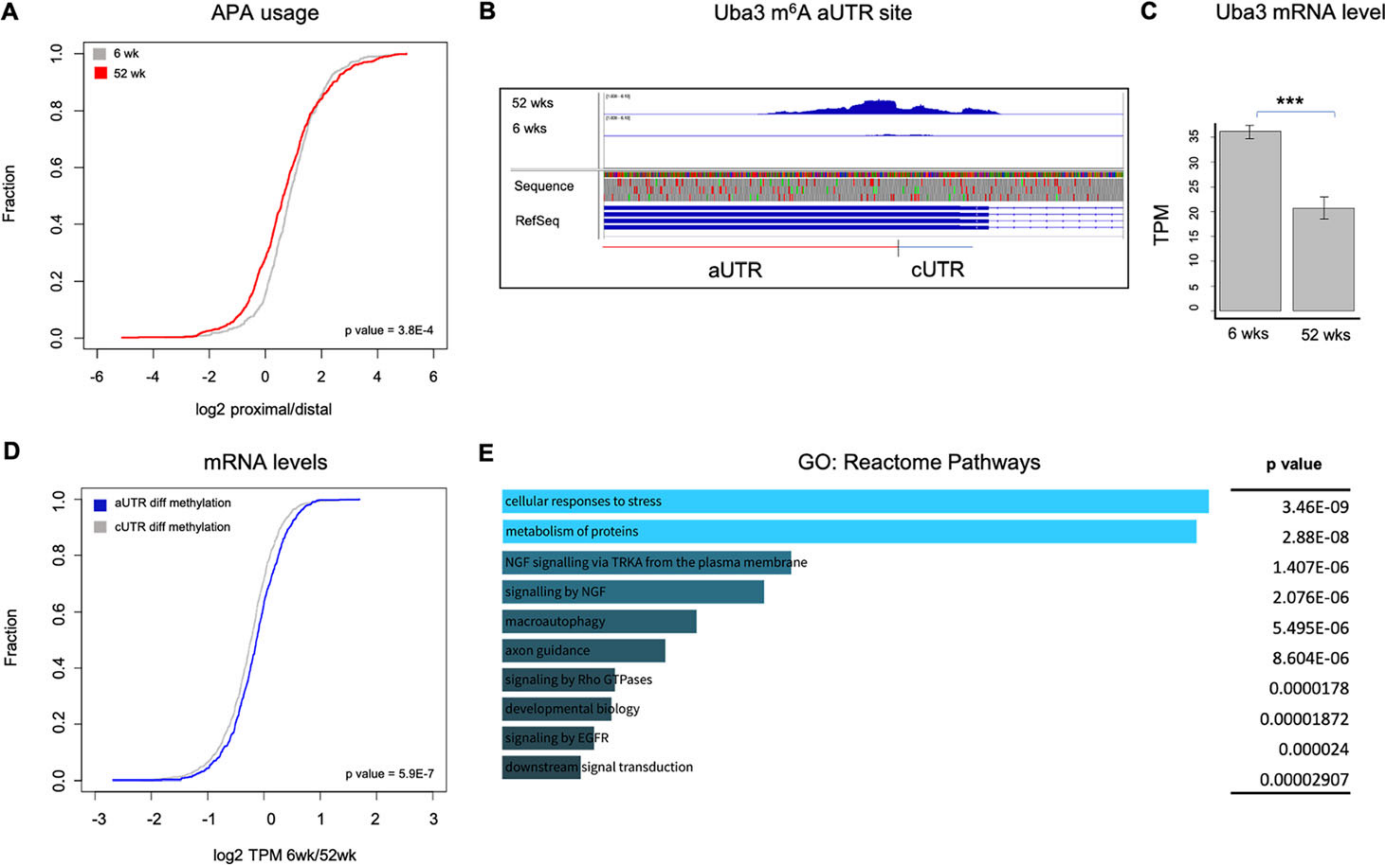
Alternative polyadenylation usage and m^6^A methylation in the aging brain. **a**, **d** Cumulative frequency plot showing the preference for proximal or distal 3′ UTR usage for genes at the 6-week and 52-week developmental stages. At 52 weeks, there is a global decrease in the log2 proximal/distal ratio compared to 6 weeks, indicating that genes prefer distal 3′ UTRs. **b** Differentially hypermethylated genes at 52 weeks preferentially display a m^6^A methylation peak in the alternative UTR (aUTR) compared to the canonical UTR (cUTR). IGV screenshot showing differential methylation in the aUTR of Uba3 at 52 weeks and not in 6 weeks. **c** Bar graph showing a highly significant decrease in the mRNA level of Uba3 at 52 weeks compared to 6 weeks. **p value < 0.01. **d** Cumulative frequency plot showing the correlation between APA usage and mRNA levels in cUTR differentially methylated genes compared to genes that display differential methylation in the aUTR. Differentially methylated genes with methylation in the aUTR have a larger log2 TPM 6wk/52wk ratio compared to genes that show cUTR differential methylation at 52 weeks. This indicates that aUTR differential methylation correlates with a markedly more significant decrease in mRNA levels compared to genes that are differentially methylated in the cUTR at 52 weeks. **e** Gene ontology analysis of enriched reactome pathways of genes that show aUTR differential methylation at 52 weeks. These pathways would be affected in the aging process

Given that m^6^A sites are enriched in the 3′ UTR and that recently it has been shown that alternative polyadenylation regulates cellular senescence [40], we next asked whether differential m^6^A methylation and differential UTR usage affects the aging process. 3′ UTRs generated by alternative polyadenylation affect gene expression by impacting both mRNA stability and translation. Generally, distal (longer) 3′ UTRs are thought to be associated with lower gene expression compared to genes possessing proximal (shorter) 3′ UTRs [41]. First, we note a global shift toward distal UTR usage in 52-week-old mice compared to 6-week-old mice, *p* value = 3.8E−4 (Fig. 4a). We next determined if differentially methylated m^6^A sites were located in the proximal or distal UTR regions. To this end, we used APAlyzer to generate lists of canonical and alternative UTRs (cUTR and aUTR, respectively) and compared those regions with the list of differentially methylated m^6^A sites. We found that 40% of differentially methylated m^6^A sites reside within the aUTR (~ 800 m^6^A methylation sites) (Additional file 7: Table S6 shows a list of transcripts with methylation in the alternative UTR). For example, Uba3, which is involved in the protein neddylation pathway, strongly expresses the aUTR form and harbors m^6^A methylation there in 52-week-old mice. In comparison, the 6-week-old mice do not show methylation (Fig. 4b), and Uba3 mRNA expression is significantly downregulated in 52-week-old mice compared to 6 weeks (Fig. 4c). Furthermore, compared to transcripts that contain m^6^A methylation in the cUTR, transcripts exhibiting m^6^A methylation in the aUTR have significantly less mRNA expression at 52 weeks relative to 6 weeks, *p* value = 5.9E−7 (Fig. 4d). Interestingly, these genes that have m^6^A in their aUTRs are involved in the metabolism of proteins, protein modification, stress, and autophagy pathways (Fig. 4e), processes that are known to be involved in the aging process. Altogether, these results suggest that m^6^A could utilize different types of UTRs to regulate processes involved in aging.

### Altered m^6^A methylation regulates protein expression in Alzheimer’s disease mouse model

**Fig. 5:**
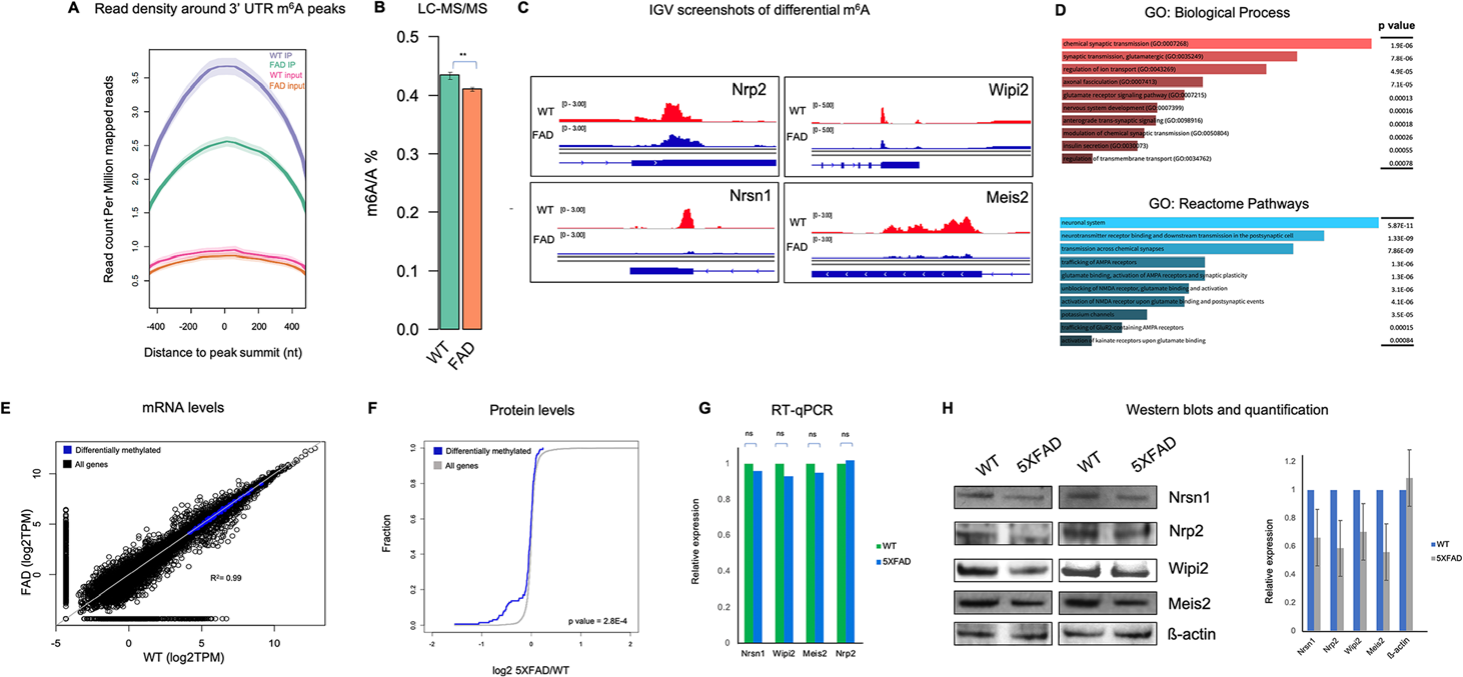
Alteration of m^6^A in the mouse model of Alzheimer’s disease. **a** Normalized read density around 3′ UTR m^6^A peaks in WT and 5XFAD. There is less m^6^A read density in 5XFAD compared to WT. Input serve as background. **b** LC-MS/MS m^6^A quantification, showing significantly higher m^6^A levels in WT compared to 5XFAD. **c** IGV screenshots showing hypomethylation in FAD compared to WT in the 3′ UTR of four representative genes. **d** Top gene ontology biological processes and reactome pathway terms of genes that show hypomethylation in FAD compared to WT. **e** Scatter plot showing no change in mRNA levels of differentially methylated genes (shown in blue) between FAD and WT. **f** Cumulative frequency plot showing the correlation between differential methylation and protein levels. Differentially hypomethylated genes in FAD show a smaller log2 FAD/WT compared to all genes. This indicates that hypomethylated genes in FAD correlate with less protein levels. **g** RT-qPCR validation, showing no significant difference in mRNA levels for the four representative genes. The results were normalized to ß-actin house-keeping gene. **h** Western validation and quantification using ImageJ, showing higher levels of protein for those four representative genes in WT compared to 5XFAD

Since we show m^6^A to be involved in the aging process, we next determined whether m^6^A plays a role in neurodegenerative disorders, such as Alzheimer’s disease. To explore this, we focused our efforts on characterizing the m^6^A methylome in 6-month-old familial Alzheimer disease mice (5XFAD) compared to age-matched wildtype (WT) control mice. The 5XFAD mouse model of AD expresses high levels of Aβ42 and at 4 months displays amyloid pathology and cognitive deficits [42], two main characteristics of the disease. Furthermore, 5XFAD mice develop neuron loss, which is not observed in most other hAPP and hAPP/PS1 AD models. We specifically chose the 5XFAD line as it is generated through co-integration of transgenes bred as a single allele, and the mice develop a rapid onset phenotype which recapitulates many AD phenotypes. Furthermore, we chose 6-month-old mice because many AD phenotypes are present in the mice at that age, including the formation of plaques, neuronal loss, gliosis, synaptic loss, and cognitive impairment [42]. Eight thousand two hundred sixty-four and 7490 m^6^A peaks were detected in WT and 5XFAD mice, respectively, with a clear enrichment of m^6^A in WT 3′ UTR sites compared to 5XFAD (Fig. 5a). LC-MS/MS confirmed an increase in m^6^A in WT poly (A) + RNA compared to 5XFAD (Fig. 5b). Interestingly, we observe an 8% increase and 4% decrease in FTO and METTL3 levels respectively in 5XFAD compared to WT mice in our RNA-seq data and in the proteomic data obtained from [43] (all other m^6^A players do not show significant changes) (Additional file 8: Table S7). A differential analysis of the m^6^A peak transcriptomes yielded 120 transcripts containing less 3′ UTR m^6^A methylation in 5XFAD mice compared to WT (Fig. 5c) (Additional file 8: Table S7). Interestingly, a gene ontology analysis showed that these differentially methylated genes are involved in biological processes that would be affected in the progression of Alzheimer’s, for example, synaptic transmission, regulation of ion transport, and axonal fasciculation. Reactome pathways are enriched for the neuronal system, neurotransmitter receptors, postsynaptic signal transmission, and protein-protein interactions at synapses (Fig. 5d).

To gain insight into the function of m^6^A on these transcripts, we first asked whether differentially methylated transcripts showed a change in mRNA levels in 5XFAD mice compared to control. To this end, we did not observe any changes in transcripts per million mapped reads (TPM) between 5XFAD mice and WT, suggesting that m^6^A is not a major regulator of mRNA levels in these transcripts (Fig. 5e). Next, we looked at whether m^6^A is associated with differential protein levels. Using the proteomic data that employed tandem mass tag mass spectrometry to deeply profile the AD brain proteome in 5XFAD mice [43], we observed that there is an association between differential m^6^A levels and protein levels. Many differentially marked transcripts correlate with decreased protein expression in 5XFAD compared to WT mice, *p* value = 2.8E−4 (Fig. 5f). In particular, many lowly expressed proteins in 5XFAD compared to WT show differential m^6^A methylation on their transcripts, suggesting that m^6^A plays a significant role in regulating protein levels in 5XFAD. We confirmed these findings by performing RT-qPCR (Fig. 5g) and Western blotting on four selective targets (Fig. 5h) (Additional file 1: Fig. S5), validating what we observed bioinformatically. For example, we observed a decrease in m^6^A methylation in the 3′ UTR of the homeobox protein MEIS2 which correlated with a decrease in protein level in 5XFAD mice (Fig. 5h), but no significant change in mRNA levels (Fig. 5g). Loss of MEIS2 has been shown to cause delayed motor development and learning disability [44], suggesting m^6^A regulation may be an important mechanism to guard against AD phenotypes. Altogether, we show that a decrease of m^6^A in the context of 5XFAD is associated with decreased levels of proteins that are strongly implicated in AD-associated pathways.

### The m^6^A pathway modulates the neuronal toxicity associated with Alzheimer’s disease

**Fig. 6:**
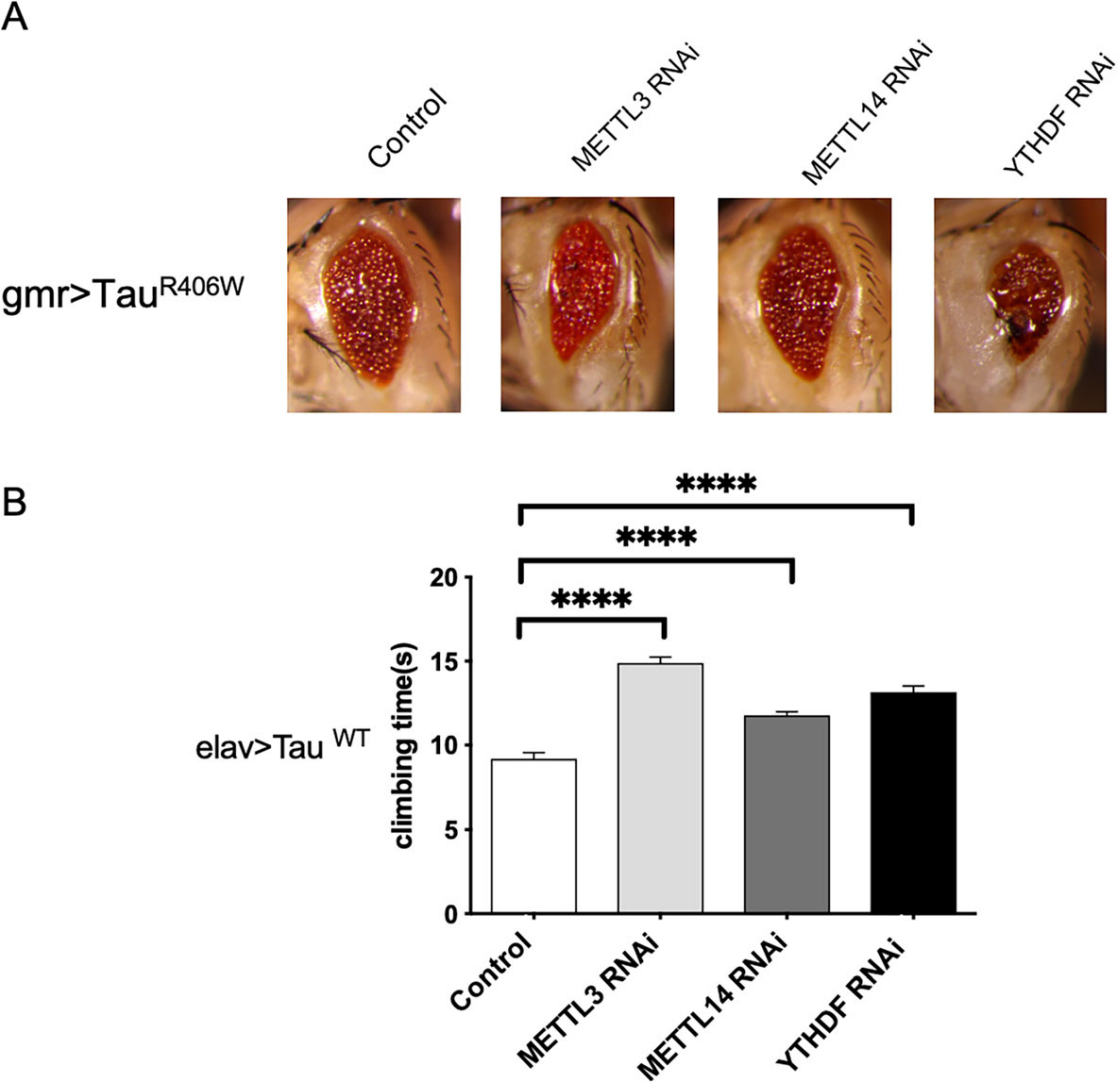
Characterizing the role of m^6^A pathway in the molecular pathogenesis of Alzheimer’s disease using the fly model. **a** Eye phenotype following the knockdown of *Drosophila* METTL3, METTL14, and YTHDF on the Tau^R406W^ background. In each case, following knockdown, eye phenotype is aggravated compared to control. **b** Column graph showing climbing times of these flies compared to control. Knockdown of the components of m^6^A pathway resulted in increased climbing times compared to control. ****p value < 0.0001

To further understand the role of m^6^A in Alzheimer’s, we investigated the effect of the m^6^A modification on Tau toxicity. In order to achieve this, we employed a *Drosophila* transgenic AD model that specifically expresses the human Tau gene with the R406W mutation in the eye using the eye-specific *gmr-GAL4* driver. Using the fly model allows us to easily determine the effects of m^6^A players in Alzheimer’s in a facile, fast, qualitative manner by visualizing changes in eye phenotype. To understand the effect of the m^6^A pathway on this AD fly model, we crossed *Drosophila* orthologs of METTL3, METTL14 (m^6^A writers), and YTHDF (m^6^A reader) RNAi flies with the AD fly (there is only one ortholog of the YTHDF proteins in *Drosophila*). We found that the eye phenotype is enhanced in all three lines compared to control (Fig. 6a), suggesting that the loss of m^6^A enhanced Tau toxicity. We then performed the climbing behavior assay to further understand the effect of m^6^A on neurodegeneration. We observed that the offspring of each of the RNAi lines crossed with Tau flies displayed an increased climbing time compared to the Tau control, suggesting that the former have more severe locomotive defects as a result of loss of METTL3, METTL14, or YTHDF (Fig. 6b). These genetic analyses together suggest an important role of m^6^A in modulating AD pathogenesis.

## Discussion

Our analysis presented here reveals roles for m^6^A in regulating neurodevelopment, aging, and Alzheimer’s disease. We find that in the mouse brain, m^6^A exerts its function in both a spatial and temporal manner. Overall, we show that differential m^6^A methylation is associated with a change in mRNA levels. We also determined a link between differential m^6^A sites and alternative untranslated region usage in transcripts involved in the aging process pathways. We extended on this observation by determining a role for m^6^A in regulating protein levels of Alzheimer disease-associated transcripts.

m^6^A levels are most prevalent in 2-week-old and 52-week-old (aged) mouse brains compared with the lowest levels being detected in 4 and 6-week-old (adolescence) mouse brains. We postulate that the observed increase in m^6^A methylation that occurs early and later in mouse brain development correlates with the most profound gene expression changes that occur at those stages, while gene expression stabilizes through adolescence and adulthood [45]. Furthermore, we find that more genes are hypomethylated than hypermethylated at 6 weeks compared to 2 weeks. The differential methylation has a more marked effect on steady-state mRNA levels than in the consistently methylated genes, and these genes are involved in distinct processes. Even though m^6^A is known to affect many aspects of RNA metabolism, as also evidenced in this work, our data suggests that the main function of m^6^A in early to adolescent brain development is to regulate steady-state mRNA levels to ensure proper development at each stage. However, we also show that m^6^A is associated with exon inclusion events across neurodevelopment. Other studies have mapped m^6^A in postnatal mouse cerebellum showing that m^6^A is important for cerebellar development by modulating gene expression of cell-fate determining genes [26]. In particular, they reveal a similar number of m^6^A sites from 1 to 8 weeks after birth, with a large majority of sites being common. Our present work suggests m^6^A has a more pivotal temporal role in regulating neurodevelopment and has a stronger impact early and later in development. Besides determining the temporal effect of m^6^A in mouse brain development, this study provides a database of m^6^A landscapes from four different brain regions, revealing expression mediated m^6^A-specific methylation in the mouse cerebellum, hypothalamus, and hippocampus. Interestingly, genes that are required for the development of specific tissues tend to be marked with m^6^A and have higher mRNA levels in that tissue compared to other tested tissues. Another study has shown specific m^6^A methylation in both cortex and cerebellar tissues in mouse and found distinct GO terms between the two tissues [24]. However, they also showed that these specifically methylated mRNAs show no change in expression between the two tissues and did not postulate a function for the spatial-specific m^6^A methylation. Our analyses suggest that the development of particular brain regions is driven in part by m^6^A methylation regulating mRNA steady levels. In addition, we found that m^6^A methylation is more prevalent in humans compared to mice at both adolescent and older time points, as did an earlier study did show more m^6^A methylation in human than in mouse during cortical neurogenesis [27]. Similar to their findings, we observed a high degree of conservation between the two species. This suggests an important function/s for m^6^A in brain aging, even more so in humans than in mice.

m^6^A is known to be enriched around stop codons and in the 3′ UTR and can therefore influence mRNA expression. Importantly, brain transcripts preferentially use distal polyA sites [46], and a recent study by Chen and colleagues has shown that distal polyA sites, resulting in the lengthening of 3′ UTR and reduced gene expression, are an important mechanism in regulating cellular senescence [40]. In this study, we find that there is a global shift toward distal 3′ UTRs in aged mice, and we discovered that differentially hypermethylated genes in aged mice display a propensity to be methylated in the alternative (distal) 3′ UTR. This has a significant impact on gene expression on these temporally differentially methylated genes. This is interesting as a previous study reported that the majority of m^6^A sites in the mouse brain are located in long last exons and suggest this may inhibit proximal polyadenylation [47]. However, our data suggests that lower mRNA expression correlates with distal m^6^A methylation. Thus, we postulate that the preferential location of m^6^A sites in the distal UTR functions to mark the transcript for degradation.

A large number of studies have shown that m^6^A RNA methylation is associated with human tumor diseases, such as breast cancer and lung cancer [48–50]. There is little known regarding the specific role, if any, of m^6^A in neurodegenerative and neuropsychiatric diseases, yet there is strong evidence to suggest a fundamental role for m^6^A in these diseases. For example, m^6^A machinery, but not m^6^A itself, has been implicated in some neurodegenerative diseases: single nucleotide polymorphisms in FTO have been implicated in many neuropsychiatric diseases [51]. Furthermore, FTO, and indeed m^6^A, may also be associated with Parkinson’s disease (PD), as dopaminergic signaling is negatively affected upon the inactivation of FTO [52]. More recently, another study looked more closely into the role of m^6^A in PD [53]. They modeled the disease in rats and PC12 cells and found a reduction of m^6^A modification. Further, a recent study undertook a cursory look into m^6^A in Alzheimer’s [54]. They found genes that were both hyper- and hypomethylated in APP/PS1 AD mice compared to control mice and that METTL3 was upregulated in the AD mouse. Those genes were involved in the presynaptic membrane, the postsynaptic membrane, and synaptic growth. They suggest that m^6^A may indeed be involved in AD but offer no mechanism or functional insights. In contrast, another recent study by [55] showed METTL3 was downregulated in the hippocampus of AD brains. Presently, we observed that 5XFAD mice display a reduction of m^6^A methylation in AD-associated genes, a mild decrease in METTL3, and an increase in FTO at both the mRNA and protein levels. We show that the loss of methylation is associated with reduced protein levels in AD, and we suggest this may be a factor in the occurrence of AD. We find the top GO term to be chemical synaptic transmission (GO:0007268). This is interesting as m^6^A is known to play an important role in synapses [56, 57], and changes in synaptic function are a major player in the progression of AD [58, 59]. Furthermore, our results were validated in vivo using an AD fly model. Knocking out m^6^A modifiers resulted in exacerbation of the AD phenotype and a decrease in motor function. We have provided strong evidence that a reduction of m^6^A plays a role in the occurrence of AD. In future studies, it would be interesting to determine the m^6^A landscape before, during, and at the height of the disease in both mice and human tissue.

## Conclusions

In summary, here we report novel insights into how m^6^A RNA methylation regulates neurodevelopment, aging, and Alzheimer’s disease. Our results show that m^6^A methylation is most prevalent early and later in neurodevelopment when the most profound changes in gene expression occur, and we also show that spatial-specific m^6^A methylation is present in mRNAs associated with tissue-specific development. We provide the first link of m^6^A regulating aging. We show that m^6^A is preferentially located within alternative UTR regions in aging and is associated with a strong decrease in mRNA expression. In addition, we provide the first genetic evidence that loss of m^6^A writers and readers could modulate the molecular pathogenesis of Alzheimer’s disease. We further show a novel role for m^6^A in regulating protein levels of Alzheimer’s disease-associated transcripts. Altogether, m^6^A is clearly an important modification in the post-transcriptional regulation of neurodevelopment, aging, and neurodegeneration.

## Methods

### Animal care

Control WT mice (C57BL/6J, Jackson Laboratory, Bar Harbor, ME, stock # 000664) and 5XFAD mice (generated on the same background, C57BL/6J 5XFAD, available from Jackson Laboratory, stock # 034840) were housed, maintained, and euthanized according to the Emory University Institutional Animal Care and Use Committee guidelines.

### RNA isolation, m^6^A-IP, and m^6^A-seq

Mice were sacrificed by cervical dislocation, and the cortex, hippocampus, hypothalamus, and cerebellum were dissected. The tissues were dissolved in TriReagent (Thermo Fisher, Waltham, MA) using a mortar and pestle and then total RNA was extracted according to the manufacturer’s instructions. Initially, m^6^A-IP reactions were performed on two biological replicates of 2-week, 6-week, and 52-week-old mice. To expand the dataset, m^6^A-IP reactions were performed on a further two biological replicates of those time points and on three biological replicates of 4-week and 26-week-old mice according to the protocol forwarded by Zeng et al. [60]. Briefly, total RNA was fragmented to 100–150 nucleotides using RNA Fragmentation Reagents (Thermo Fisher, AM8740) by incubating at 70° for 5 min 30 s. Fragmented RNA was purified using ethanol precipitation overnight and then incubated with 2.5 μg of anti-N6-methyladenosine antibody at 4 °C for 2 h, followed by the addition of 30 μl of Dynabeads™ Protein A for Immunoprecipitation (Thermo Fisher, 10002D), and incubated for a further 2 h. Beads were washed four times with IPP buffer (150 mM NaCl, 0.1% NP-40, 10 mM Tris-HCl, pH 7.4), and immunoprecipitated RNA was recovered through elution with 2.6 mg/ml N6-methyladenosine 5%-monophosphate sodium salt (Sigma M2780) followed by ethanol precipitation. For m^6^A-seq, a cDNA library was constructed using the SMARTer Stranded Total RNA Sample Prep Kit (Takara 634861). Total RNA that was not subjected to immunoprecipitation was also used to RNA-seq libraries to serve as an input.

### PCA plots

Input BAM files were subtracted from IP BAMs using bamCompare from the deeptools package [61]; compressed numpy array files were then generated using the multiBamSummary command and PCA plots were generated using plotPCA command, from the deeptools package.

### m^6^A peak calling, differential peak calling, and motif analysis

To identify regions in which m^6^A modifications occur, we used the peak calling algorithm MACS2 (version 2.1.0.20140616) [62] on m^6^A-seq, using the corresponding RNA-seq as input (background). Studies have shown that MACS2 is a suitable tool to analyze MeRIP-seq data. For example, work by Liu et al. [63] compared MACS2 and exomePeak and showed a significant correlation between the results obtained on MeRIP-seq datasets using the two techniques. And a more recent study by McIntyre et al. [64] drew the same conclusions, stating that most people use MACS2 to analyze MeRIP-seq and they found it to be a reliable tool. In fact, these authors found MACS2 outperforms exomePeak. Another study by Anatanaviciute et al. [65] also shows that MACS2 was a better performer than exomePeak. The MACS2 callpeak function was run with the following parameters: –nomodel,–extsize 150, -p 5e-2, and –g mm. Therefore, candidate m^6^A peaks were identified as an enrichment of reads upon pull down with the m^6^A antibody compared to background. To identify differential peaks, the MACS2 differential binding events program (bdgdiff) with parameters -g 20 and -l 120 was employed. The consensus sequence motifs enriched in m^6^A peaks were identified by using MEME (version 5.1.1) [66]. The integrative genomics viewer (IGV) tool was used for visualization of m^6^A peaks along the whole transcript [67].

### Alternative splicing and m^6^A

Poly (A) + enriched mRNA-seq libraries were deeply sequenced. Alternative splicing events were determined using rMATs [68]. m^6^A sites were overlapped with alternative splicing events using “subsetByOverlaps” command in the GenomicRanges package in R.

### m^6^A quantification by LC/MS-MS

Poly(A) mRNA was isolated from total RNA using NEBNext poly(A) mRNA magnetic isolation according to the manufacturer’s protocol. One hundred fifty nanograms of mRNA was digested by nuclease P1 (1 U) in 25 μl of buffer containing 100 mM NH4OAc at 42 °C for 2 h. Subsequently, NH_4_HCO_3_ (1 M, 3 μl) and alkaline phosphatase (1 U) were added and further incubated for another 2 h at 37 °C. The samples were then filtered using a 0.22-μm PVDF filter (Millipore, P. N.: SLGVR04NL) and 5 μl of the solution was injected into the LC-MS/MS. HPLC-MS/MS was carried out by reverse-phase ultra-performance liquid chromatography on an Agilent ZORBAX Eclipse XDB-C18 column (Rapid Resolution HT, 50 × 2.1 mm (P.N. 927700-902), equipped with a ZORBAX Eclipse XDB-C8 guard column (Column: P.N. 821125-926, Cartridges P.N. 820555-901), eluted with buffer A (0.1% formic acid in H_2_O) and buffer B (0.1% formic acid in methanol) with a flow rate of 0.5 ml min^−1^ at 35 °C with a 2–25% gradient in 4.5 min, with online mass spectrometry detection using Agilent 6410 triple-quadrupole (QQQ) LC mass spectrometer in multiple reaction monitoring (MRM) positive electrospray ionization (ESI) mode. The nucleosides were quantified using the nucleoside to base ion mass transitions of 282.1 to 150.1 (m^6^A), 268.0 to 136.0 (A).

### Correlation analysis between RNA m^6^A methylation level and RNA expression level

The heatmaps showing the correlation between m^6^A and TPM values were plotted using the pheatmap package in R.

### Correlation analysis between RNA m^6^A methylation and APA usage

APAlyzer [69] was used to generate lists of canonical and alternative UTRs (cUTR and aUTR respectively). A pre-built reference file for the mouse genome (mm9) was used which contained the APA regions (refUTRraw), IPA regions (dfIPA), and 3′-most exon regions (dfLE). Genomic ranges were created and the intersect between those regions was found using “subsetByOverlaps” command in the GenomicRanges package in R.

### RNA-seq data processing, reads mapped, and mRNA quantification

Raw sequencing reads were preprocessed to remove adaptor and poor-quality bases using Trimmomatic (version 0.38) with the following parameters: TruSeq3-PE.fa:2:30:10 LEADING:20 TRAILING:20 SLIDINGWINDOW:4:15 MINLEN:15. Quality control was then performed using FastQC software (version 0.11.4). Surviving high-quality reads were mapped against the mouse genome (mm9) using Bowtie 2 software (version 2.2.6), allowing no mismatches in the seed region and tophat-2.1.1 [70]. Duplicate reads were then removed used samtools. TPM (transcripts per million mapped reads) were calculated using TPMcalculator tool to quantify mRNA abundance [71]. The RNA-seq data served as input for the m^6^A-seq.

### Gene ontology analysis

The Enrichr tool [72] was used to perform the GO analysis using default parameters. Bar plots were generated based on the enriched GO terms using Prism software. The length of the bar represents the significance of that specific term based on log_10_(*P*value), and the lighter the color bars means higher significance. A full list of all selected terms of the biological process, cellular components, and molecular functions category are provided in Supplemental tables.

### Characterization of m^6^A peak distribution patterns

The normalized m^6^A peak read density plot was generated using NGSPLOT (v2.63) [73], using a config file containing the genomic coordinates of 3′ UTR m^6^A sites.

### Reverse transcription and qPCR using Taqman assay

Five micrograms of total RNA was treated with DNAse I (Promega), then reverse transcribed, using 200 U Superscript III (Invitrogen) and 50 μM of oligo dT primers (Invitrogen), according to the manufacturer’s instructions. cDNA was diluted 1:5 in nuclease-free water. Triplicate TaqMan qPCR reactions (20 μl total volume) were performed using 50 ng of the diluted cDNA, 1x TaqMan Gene Expression assays, and 1x TaqMan Universal PCR Master Mix II (Thermo Fisher Scientific). All TaqMan Gene Expression assays are from Thermo Fisher Scientific and are listed here: Nrsn1 (Mm00494159_m1), Wipi2 (Mm00617842_m1), Nrp2 (Mm00803099_m1), Meis2 (Mm00487748_m1), and ß-actin (Mm01210323_m1) were used for normalization.

### Western blotting

Five milligrams of cortical 5XFAD and WT tissue were homogenized using a pestle in 300 μl of RIPA buffer; subsequently, another 300 μl of buffer was added and incubated with rotation at 4 °C for 2 h. BCA assay was performed to measure protein concentration according to the manufacturer’s protocol (ThermoFisher Scientific). Ten micrograms of protein was loaded per sample on a 4–12% Bis-Tris.

Nupage™ gel (Invitrogen) was transferred on a nitrocellulose membrane and probed with antibodies against Nrsn1 (ab237502), Wipi2 (ab105459), Nrp2 (ab185710), Meis2 (ab73164), and ß-actin (A3853).

### *Drosophila* genetics and climbing assay

The gmr*-*GAL4 and UAS-TRiP lines were obtained from the Bloomington Stock Centre (Bloomington, IN, USA). All crosses were grown on standard medium at 25 °C. Targeted expression of human mutant Tau^R406W^ in the fly eye presents highly uniform eye degeneration of reduced eye size accompanying with rough eye surface. Crosses between Tau and candidate genes were performed to investigate modifiers with enhancement or suppression effect in view of eye phenotype under light microscopy. More specifically, RNAi lines of the m^6^A modifiers were crossed with Tau^R406W^ mutant flies. After 5 days, eye phenotypes of co-expression of Tau with the modifiers were assessed using light microscopy and confirmed with scanning electron microscopy.

To assess motor function of these flies, a climbing assay was performed. Groups of ten 5-day-old flies were transferred into 1.25-cm-diameter and 28-cm-height plastic tubes with 1-h incubation at room temperature to wake from anesthesia and acclimatize the new environment. The arrival time of the fifth fly at the 15-cm finish line was collected and analyzed. Three trials were repeated for each group. For statistical analyses, comparisons were made using Student’s *t* test.

### Scanning electron microscopy

Whole flies were dehydrated in increasing concentrations of ethanol (25, 50, 75, and 100%), then incubated for 1 h with hexamethyldisilazane (Electron Microscopy Sciences, Hatfield, PA). After removing the hexamethyldisilazane, the flies were dried overnight in a fume hood and subsequently analyzed using the Dual Stage Scanning Electron Microscope DS 130F (Topcon, Tokyo, Japan).

### Quantification and statistical analysis

The Wilcoxon test was used for the cumulative frequency distribution plots. A Student *t* test to determine significant changes in mRNA levels and protein levels and in the climbing assay analyses. All statistical analyses were conducted using the R package. *p* values are indicated in figures and figure legends and text.

## Supplementary Information

**Additional file 1: Fig. S1.** Numbers and comparisons of genes containing m^6^A in the 4 brain regions (cortex, cerebellum, hippocampus and hypothalamus) at 2-week, 6-week and 52-weeks post birth in mice. **Fig. S2.** PCA plots showing reproducibility amongst replicates and differences between samples. **Fig. S3.** UCSC screenshots showing m^6^A associated exon inclusion events. **Fig. S4.** Integrative Genomics Viewer (IGV) screenshots showing tissue-specific m^6^A methylation. **Fig. S5.** Uncropped full Western blots.

**Additional file 2: Table S1.** Number of uniquely mapped reads for each time point and brain region for both m^6^A IP and inputs.

**Additional file 3: Table S2.** List of called peaks at each time point and brain region.

**Additional file 4: Table S3.** List of differentially methylated genes between 2 weeks and 6 weeks in the 4 brain regions.

**Additional file 5: Table S4.** List of m^6^A associated exon inclusion events across neurodevelopment.

**Additional file 6: Table S5.** List of tissue-specific differentially methylated genes and how they correlate with TPM levels.

**Additional file 7: Table S6.** List of differentially methylated genes between young and old in mouse and human and list of differentially methylated genes with methylation occurring in alternative 3′ UTR.

**Additional file 8: Table S7.** List of differentially methylated genes between FAD and WT and how they correlate with protein levels.

**Additional file 9:** Review history.

## Data Availability

RNA-seq and m^6^A-seq data are available from NCBI’s Gene Expression Omnibus (GEO). The accession number for all the datasets reported in this paper is GSE144032 [74]. The 5XFAD proteomic data used in this study was sourced from Bai et al. [43] and is accessible from the PRIDE database (www.proteomexchange.org): with accession numbers of PXD007974 and PXD018590. Scripts used throughout this study are accessible from Github at https://github.com/ashafik1/Shafik_et_al_codes/tree/main [75]. The codes are also available at Zenodo with DOI:10.5281/zenodo.4279626 [76]. LC-MS/MS m^6^A data is available at Figshare with DOI:10.6084/m9.figshare.13356179 [77].
