# Supplementary material for: N6-methyladenosine dynamics in neurodevelopment and aging, and its potential role in Alzheimer’s disease
Source: Genome Biol. 2021 Jan 5;22:17. doi: 10.1186/s13059-020-02249-z (PMC7786910; doi:10.1186/s13059-020-02249-z)
Supplement: Supplementary file 1 — Additional file 1: Fig. S1. Numbers and comparisons of genes containing m6A in the 4 brain regions (cortex, cerebellum, hippocampus and hypothalamus) at 2-week, 6-week and 52-weeks post birth in mice. Fig. S2. PCA plots showing reproducibility amongst replicates and differences between samples. Fig. S3. UCSC screenshots showing m6A associated exon inclusion events. Fig. S4. Integrative Genomics Viewer (IGV) screenshots showing tissue-specific m6A methylation. Fig. S5. Uncropped full Western blots. [file 13059_2020_2249_MOESM1_ESM.pdf]

Supplementary Information – Shafik et al

Supplementary Figures

Fig. S1: Numbers and comparisons of genes containing m^6A in the 4 brain regions (cortex, cerebellum, hippocampus and hypothalamus) at 2 weeks, 6 weeks and 52 weeks post birth in mice.

Fig. S2: PCA plots showing reproducibility amongst replicates and differences between samples.

Fig. S3: UCSC screenshots showing m^6A associated exon inclusion events.

Fig. S4: Integrative Genomics Viewer (IGV) screenshots showing tissue-specific m^6A methylation.

Fig. S5: Uncropped full Western blots.

Supplementary Tables

Table S1: Number of uniquely mapped reads for each time point and brain region for both m^6A IP and inputs

Table S2: List of called peaks at each time point and brain region.

Table S3: List of differentially methylated genes between 2 weeks and 6 weeks in the 4 brain regions.

Table S4: List of m^6A associated exon inclusion events across neurodevelopment.

Table S5: List of tissue-specific differentially methylated genes and how they correlate with TPM levels.

Table S6: List of differentially methylated genes between young and old in mouse and human and list of differentially methylated genes with methylation occurring in alternative 3' UTR.

Table S7: List of differentially methylated genes between FAD and WT and how they correlate with protein levels.

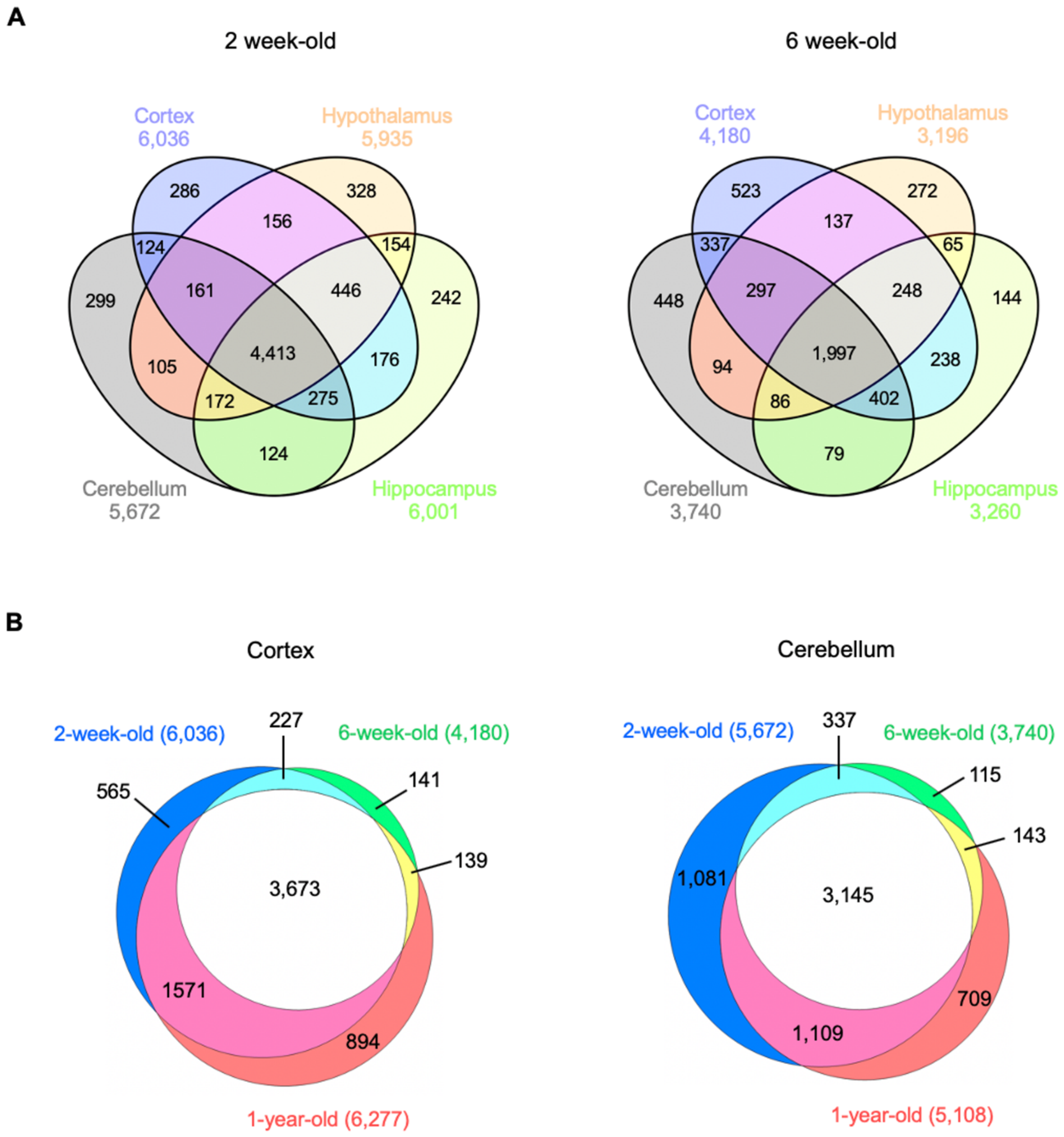

Fig. S1: (A) Comparison of genes containing m^{6}A in the 4 different brain regions at 2 weeks and 6 weeks. (B) Comparison of genes containing m^{6}A in the cortex and cerebellum between 2 weeks, 6 weeks and 52-week-old mice.

2wk (batch 1)

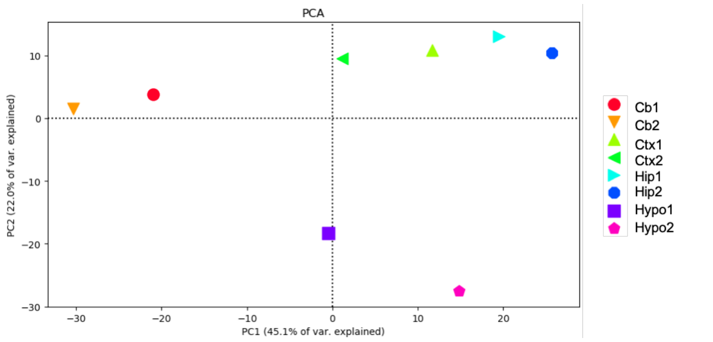

2wk (batch 2)

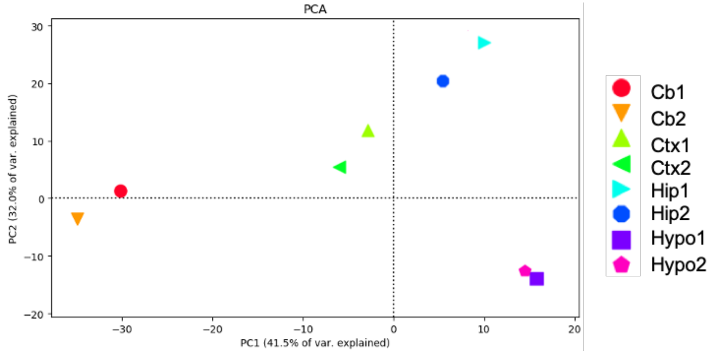

4wk

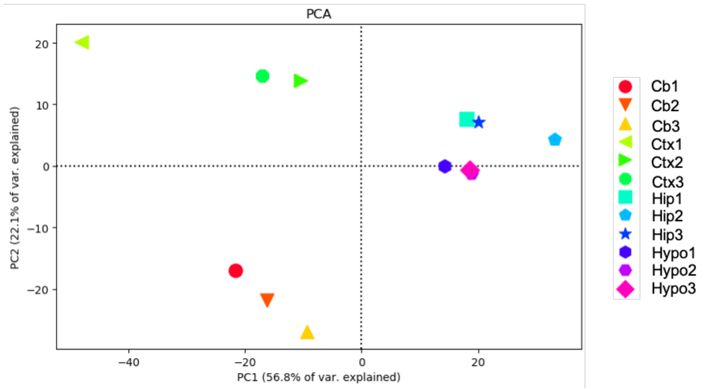

6wk (batch 1)

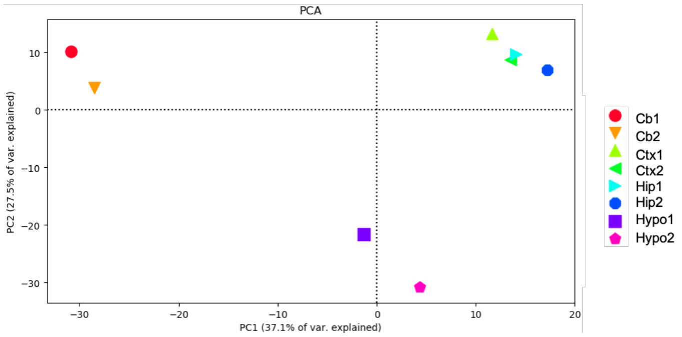

6wk (batch 2)

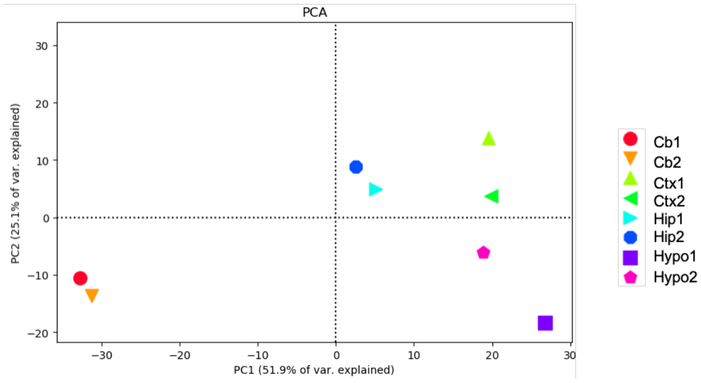

26wk

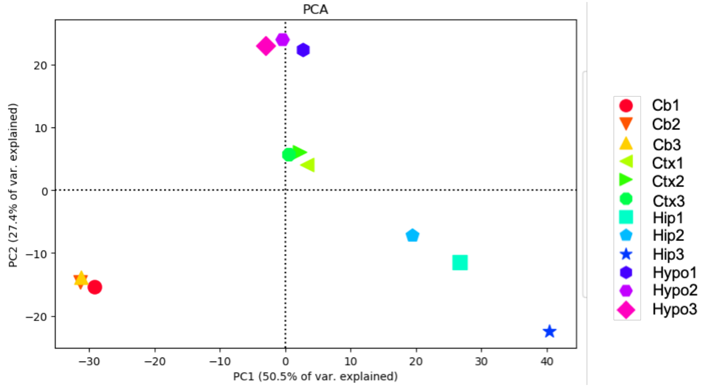

52wk (batch 1)

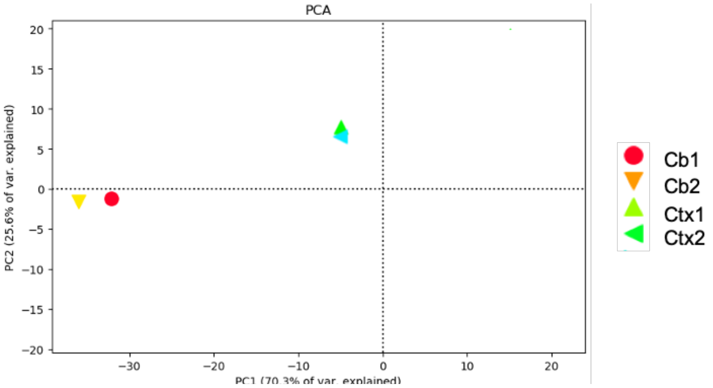

52wk (batch 2)

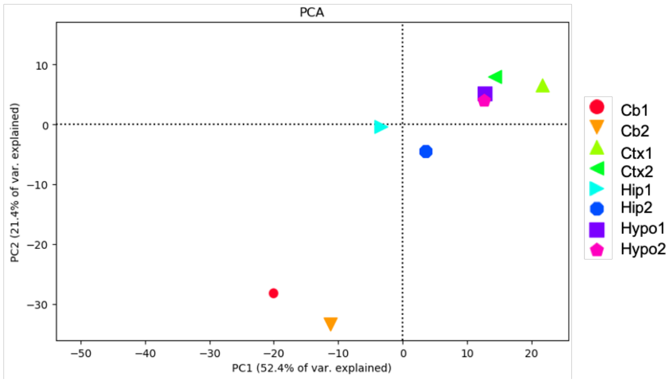

Alzheimer's

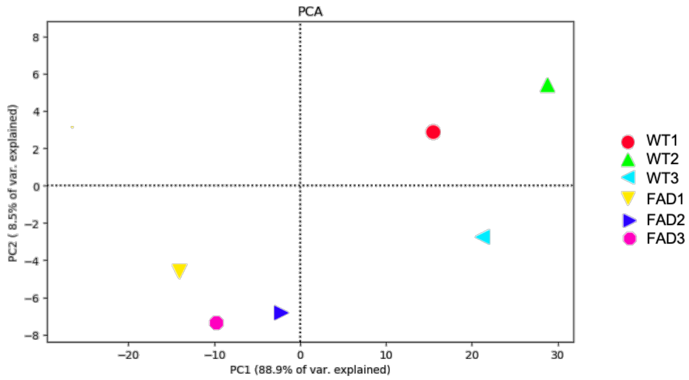

Fig. S2: PCA plots.

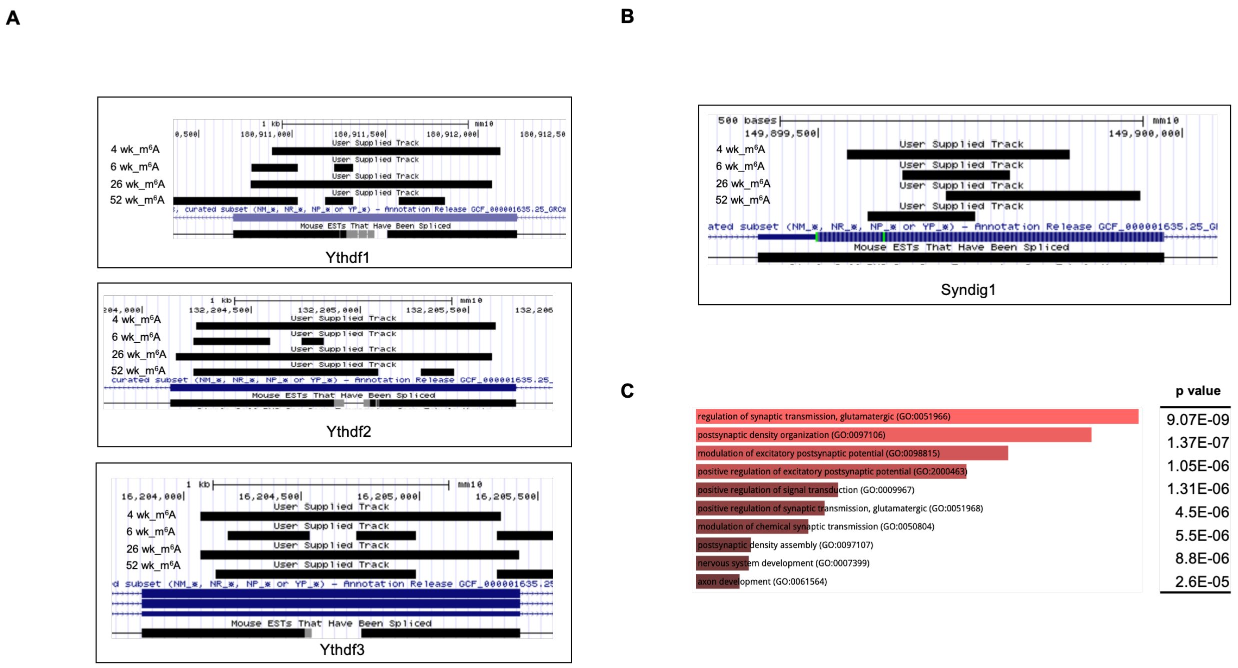

Fig. S3: UCSC screenshots showing association between m^{6}A sites (shown as black bars – user supplied tracks) and exon inclusion events (shown by the mouse ESTs that have been spliced track) (A) in the m^{6}A binding proteins YTHDF1, YTHDF2, YTHDF3, and in B) Syndig1. The rMATs analysis shows that these exons are retained in our samples (see Table S4) C) gene ontology of m^{6}A associated exon inclusion events showing those genes are involved mainly in synaptic processes.

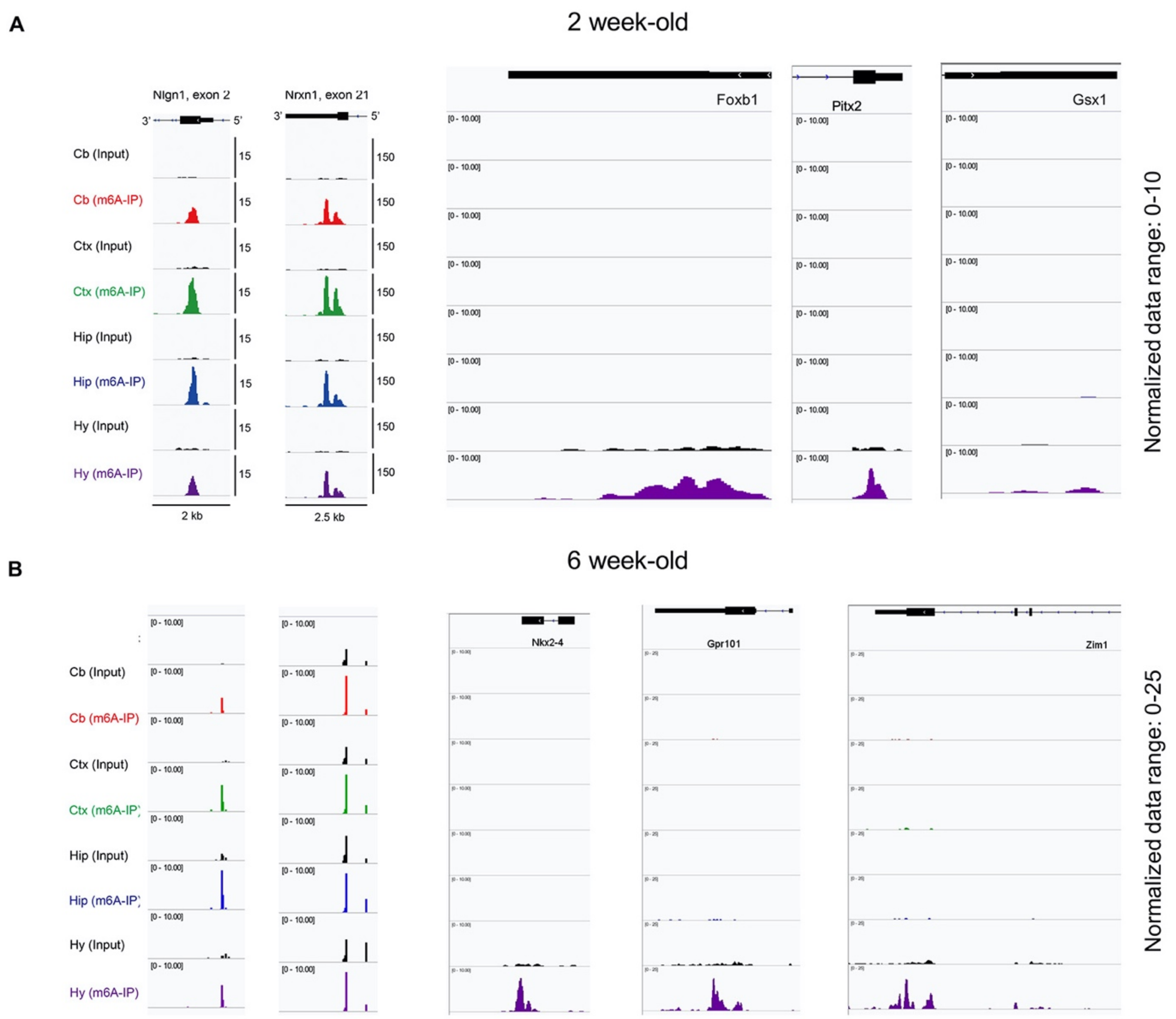

Fig. S4: Integrative Genomics Viewer (IGV) plot containing four tracks for non-IP control reads (black) and four tracks for m^{6}A-IP reads (color) at specific loci. (A) Hypothalamus specific m^{6}A peaks at 2 weeks. These genes are specifically involved in hypothalamus development and (B) at 6 weeks. These genes are involved in developmental pathways. Nlgn1 and Nrxn1 loci were displayed as controls.

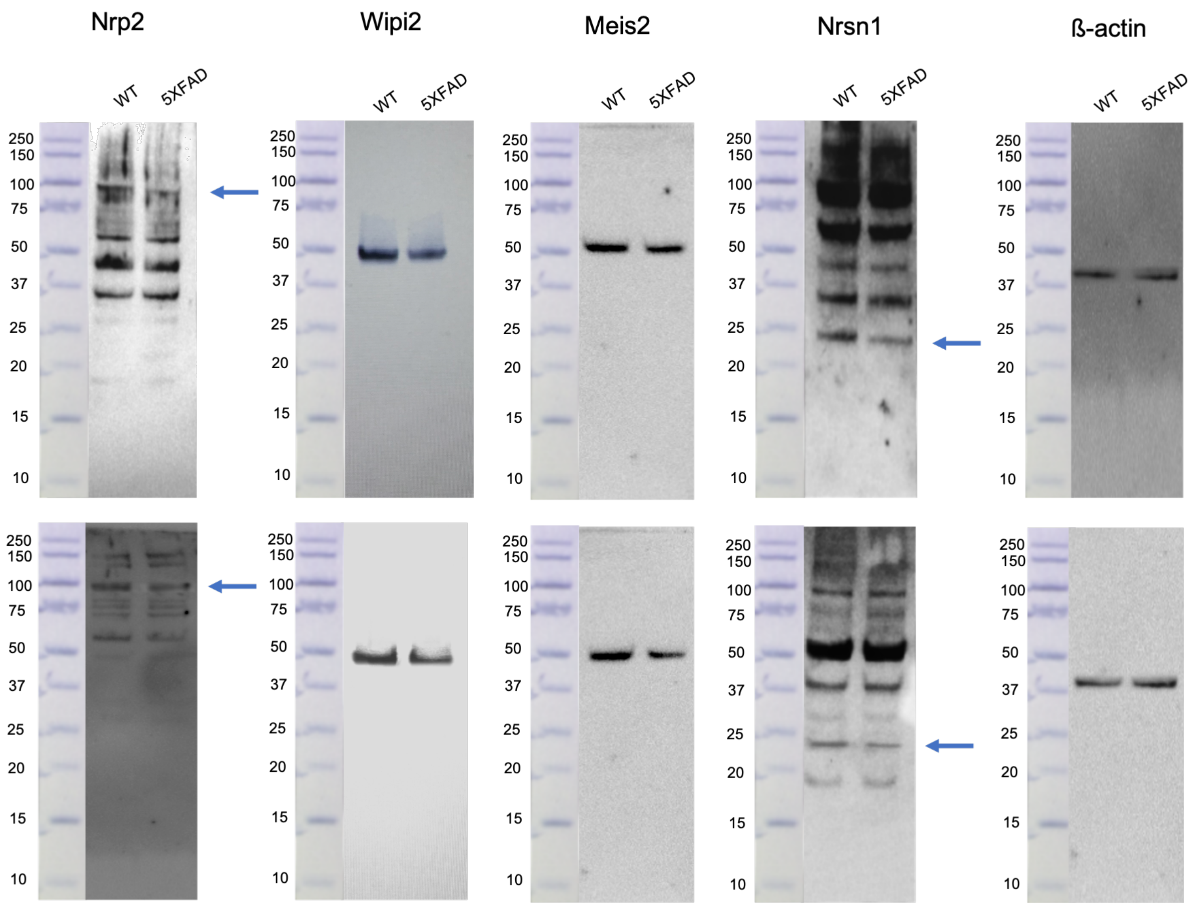

Fig. S5: Uncropped full Western blots.
