# Supplementary material for: N6-methyladenosine dynamics in neurodevelopment and aging, and its potential role in Alzheimer’s disease
Source: Genome Biol. 2021 Jan 5;22:17. doi: 10.1186/s13059-020-02249-z (PMC7786910; doi:10.1186/s13059-020-02249-z)
Supplement: Supplementary file 9 — Additional file 9: Review history. [file 13059_2020_2249_MOESM9_ESM.docx]

Review History

**First round of review**

**Reviewer 1**

**Are you able to assess all statistics in the manuscript, including the appropriateness of statistical tests used?** Yes, and I have assessed the statistics in my report.

**Comments to author:**

The manuscript entitled "6-methyladenosine dynamics in neurodevelopment and aging and its potential role in Alzheimer's disease" presents exciting new findings that link m6A to neurodevelopment, aging, and AD. The approach/methods are appropriate and the data supports the conclusions. This manuscript represents a significant advance over previously published studies and is of broad interest to others in the field, making it appropriate for publication in Genome Biology.

I have only minor concerns/suggestions:

1) it is concerning that the authors pooled six mice into "2 biological replicates." Despite the fact that the data represents 6 genomes, it would be nice to have seen a third biological replicate to enable proper estimation of the variance in the data. The inclusion of an input control was important for background correction. This approach likely overestimates the number of "true" peaks in the data.

2) it would be helpful if the p-value cutoff used was included in the text of the results section.

3) some of the language is "non-scientific." For example, use of the statement "tend to have" and stating the groups had "about 1,800 transcript" or "about 960 differential peaks" in the results section should be more exact. Please edit to include the exact numbers, as this will help the reader find the corresponding numbers on figures.

4) the authors examined 6-methyladenosine in several tissues. In some sections of the results they provide general information on all tissues before focusing on a specific tissue; however, in some cases it's not clear what tissue. For example, in figure 1 C,D, and E, it's not clear if the authors are only talking about the cerebellum data or if these findings are found using data from all tissues. If the authors could include the tissue name in the figure or text that would help the reader follow.

5) the authors refer to "correlated with a decrease in protein level in 5XFAD mice" without providing this data or a reference for this data. Please include either.

6) the authors report an "enrichment of m6A around the start, stop, and 3'UTR" and "a clear decrease in enrichment in those regions in 5XFAD mice compared to WT." However, in the figure it appears that the decrease in 5XFAD mice is not restricted to these region, as there is a decrease across all genic regions. Is there a significant decrease in any (or all) of these regions?

7) the authors state, "Loss of MEIS2 has been shown to cause delayed motor development and learning disability..." without providing the reference for this statement. This should be added.

8) the GO terms presented in figure 5C are all highly connected to AD phenotypes. Are these the top 10 GOs are a selected set? This should be stated in the legend.

**Reviewer 2**

**Are you able to assess all statistics in the manuscript, including the appropriateness of statistical tests used?** No, I do not feel adequately qualified to assess the statistics.

**Comments to author:**

In this study by Shafik et al., entitled 'N6-methyladenosine dynamics in neurodevelopment and aging and its potential role in Alzheimer's disease' the authors profiled m6A mRNA modification (by methylated RNA immunoprecipitation and sequencing, meRIP-seq) in developing and aging mouse and human brains. They further extended the story by determining the landscape of m6A in the context of an Alzheimer's mouse model. The authors suggested that RNA m6A methylation plays a role in the regulation of the aging mammalian brain by regulating mRNA expression levels of transcripts involved in aging through 3' untranslated regions (UTRs), while also playing a role in the occurrence of Alzheimer's disease by regulating mRNA translation. Finally the study includes RNAi-mediated loss of function studies of the m6A Methyltransferase complex (METTL3, METTL14) and the m6A-reader protein YTHDF2, in a fly model.

While the topic of this work is of substantial interest the overall evidence supporting the authors conclusions falls considerably short of the standard expected for publication Genome biology.

Major concerns:

1)	The authors chose only 3 time-points of mouse development and found that the m6A profiles go down and then up from 2-week to 6-week and to 1-year old. However, 1) the reliability is largely compromised by the very limited number of time points and small number of samples (two replicates) used for each timepoint; 2) the data quality, rather than real biological differences, could therefore be the main cause of theses observations.

2)	The authors use of 'number of peaks' to represent the overall m6A level is very problematic (Figure 1A), since the peak calling can be affected by many non-biological factors, including peak calling parameters, sequencing depth, rRNA ratio, etc. Actually, the overall sequencing depth of the 6-week samples are lower than the other two timepoints. The authors should at least confirm the results by either using sequencing spike-in controls, and/or Mass spec to evaluate the global m6A content of each sample. It is hard to determine whether one modification peak from meRIP-seq data is sample- or tissue-specific, because even a single parameter difference in MACS2 software will result in different number of peaks. Besides, as far as I know, the differential peak analysis based on MACS2 is not highly accurate, the authors can try to use other tools designed for m6A analysis, such as exomePeaks to identify the differential m6A peak intensity

3)	Throughout the manuscript, the authors should address and clarify 'm6A specificity' vs. 'gene expression specificity' (e.g. Figures 1D, Fig 2, etc.). My impression is that most of the 'differential m6A' described here is simply due to the relative expression of the host mRNA rather than reflecting dynamic m6A modification. Accordingly, many of the enriched GO pathways could simply from the tissue/age specific gene expression.

4)	PCA analysis should be performed to check the m6A distribution among different tissues and among brain development time points. Meanwhile, reproducibility should be checked using correlation analyses between replicates.

5)	There is no experimental validation to support the authors' conclusions. More stringent validations, including differential translation validation using western blots, and expression of reporters with 3' untranslated region (UTR) are definitely needed.

6)	m6A has been reported in regulating many post-transcriptional regulation processes, such as translation, alternative polyadenylation (APA), and alternative splicing. Splicing should be also examined and included in the results.

7)	The authors should use more specific sequencing technology to evaluate the APA events, such as PAS-seq, PolyA-seq, and PAT-seq.

8)	Page 6, line 33-34, the authors cannot use gene expression changes to indicate mRNA stability. The expression difference can be due to both transcription differences and/or RNA degradation. Besides, it is not accurate to use the gene expression to demonstrate that the m6A changes in AD resulted in RNA turnover. Transcriptional inhibition with Actinomycin D treatment should be used to study mRNA stability (or half-life).

9)	A comparative analysis of m6A genes between mouse and human should be performed to study whether the m6A genes show the same trend between mouse and human brains.

10)	For the Ribo-seq data, the authors used the publicly available Ribo-seq data of YTHDF1 and YTHDF2 knockdown samples (in HeLa cells), which is not directly relevant to their story. Ribo-seq sequencing on the AD disease model or iPSCs from AD disease sample should be conducted to check the differential translations. Moreover, the data analysis and conclusions from the YTHDF1 paper (Wang et al., Cell 2015; citation #5 in the current manuscript) have been recently called into question (Zaccara and Jaffrey, Cell 2020), and therefore not considered satisfactory for this work.

11)	The authors should pay more attention to m6A modifications of the AD-related genes (https://www.alzforum.org/genetics). Whether these genes show different m6A modifications, mRNA expression, and/or translation in AD models?

12)	Figure 5A: The conclusion of global reduction simply based on sequencing data is problematic and unconvincing. Again, the authors should try to use spike-in RNA and/or mass spec to validate this possible change.

13)	Figure 5D, and page 10, line 55, 'suggesting that m6A is not a major regulator of mRNA stability in these transcripts'. The authors should explain the discrepancy with their conclusion in Figure 1, where they found that m6A is related to the expression/stability of the host mRNA. Since AD is a age-related phenotype, the effect should be consistent.

14)	The authors should explain and justify why they chose fly for validation instead of mice here?

15)	The experimental design in the fly validation is very confusing. In figure 5, the authors found that the YTHDF1 could be the driver of the m6A-mediated phenotype, but why did they choose YTHDF2 instead in the RNAi experiment in fly?

Other minor points:

1. Figure 1B: This distribution plot looks weird. What do the START and STOP here mean? If it's start codon and stop codon, then why the 3'UTR are largely depleted while Fig 1A shows there are quite a lot 3'UTR peaks?

2. Figure 1C: I couldn't figure out from which peak set the authors got this motif? Perhaps the authors can compare the motif enrichments in different dataset to see if there is peak quality difference between samples.

3. Figure 5E: Why didn't the authors directly compare the translation efficiency between AD vs non-AD here? Which would be much more straightforward.

**Authors Response**

**Point-by-point responses to the reviewers’ comments:**

**Reviewer 1**

1)it is concerning that the authors pooled six mice into "2 biological replicates." Despite

the fact that the data represents 6 genomes, it would be nice to have seen a third

biological replicate to enable proper estimation of the variance in the data. The

inclusion of an input control was important for background correction. This approach

likely overestimates the number of "true" peaks in the data.

*This is a good suggestion. We have included 2 more time points at 4-weeks and 26-*

*weeks, whilst also having at least triplicates for each time point in the revised*

*manuscript. Furthermore, we employed a new m6A-seq protocol that allowed >3 ug*

*total RNA input. Thus, we were able to get sufficient RNA from 1 mouse for each*

*sample. m6A peaks were identified as at least a 2-fold enrichment over the input*

*control.*

2)it would be helpful if the p-value cutoff used was included in the text of the results

section.

*The p-value cutoffs have been included in the results section where appropriate.*

3)some of the language is "non-scientific." For example, use of the statement "tend to

have" and stating the groups had "about 1,800 transcript" or "about 960 differential

peaks" in the results section should be more exact. Please edit to include the exact

numbers, as this will help the reader find the corresponding numbers on figures.

*We thank the reviewer for this comment. The language in the results has been*

*amended to be more precise.*

4)the authors examined 6-methyladenosine in several tissues. In some sections of the

results they provide general information on all tissues before focusing on a specific

tissue; however, in some cases it's not clear what tissue. For example, in figure 1 C,D,

and E, it's not clear if the authors are only talking about the cerebellum data or if these

findings are found using data from all tissues. If the authors could include the tissue

name in the figure or text that would help the reader follow.

*We thank the reviewer for this comment and have amended the text and legend to*

*make this clearer.*

5)the authors refer to "correlated with a decrease in protein level in 5XFAD mice"

without providing this data or a reference for this data. Please include either.

*The reference for this data has been noted in the text.*

6)the authors report an "enrichment of m6A around the start, stop, and 3'UTR" and "a

clear decrease in enrichment in those regions in 5XFAD mice compared to WT."

However, in the figure it appears that the decrease in 5XFAD mice is not restricted to

these regions, as there is a decrease across all genic regions. Is there a significant

decrease in any (or all) of these regions?

*We thank the reviewer for this comment. Since we only detect 3’UTR differential sites, we have amended that panel of the figure to show the normalized read density around*

*called 3’UTR m6A peaks in 5XFAD and WT. The main text and figure legend have*

*been changed to reflect this.*

7)the authors state, "Loss of MEIS2 has been shown to cause delayed motor

development and learning disability..." without providing the reference for this

statement. This should be added.

*A reference for this statement has been added to the text.*

8)the GO terms presented in figure 5C are all highly connected to AD phenotypes. Are

these the top 10 GOs are a selected set? This should be stated in the legend.

*The panel is now in Figure 5D*

**Reviewer 2**

1)The authors chose only 3 time-points of mouse development and found that the m6A

profiles go down and then up from 2-week to 6-week and to 1-year old. However, 1)

the reliability is largely compromised by the very limited number of time points and

small number of samples (two replicates) used for each timepoint; 2) the data quality,

rather than real biological differences, could therefore be the main cause of theses

observations.

*We thank the reviewer for this comment. We have addressed this by including 2 more*

*time points at 4-weeks and 26-weeks, whilst also processing another batch of 2-week,*

*6-week and 52-week-old mice.*

2)The authors use of 'number of peaks' to represent the overall m6A level is very

problematic (Figure 1A), since the peak calling can be affected by many non-biological

factors, including peak calling parameters, sequencing depth, rRNA ratio, etc. Actually,

the overall sequencing depth of the 6-week samples are lower than the other two

timepoints. The authors should at least confirm the results by either using sequencing

spike-in controls, and/or Mass spec to evaluate the global m6A content of each

sample. It is hard to determine whether one modification peak from meRIP-seq data is

sample- or tissue-specific, because even a single parameter difference in MACS2

software will result in different number of peaks. Besides, as far as I know, the

differential peak analysis based on MACS2 is not highly accurate, the authors can try

to use other tools designed for m6A analysis, such as exomePeaks to identify the

differential m6A peak intensity.

*We thank the reviewer for this comment but disagree for the following reasons: 1)*

*Differences in sequencing depth are controlled for by library size normalization. 2)*

*Changes in mRNA expression due to sample differences are accounted for by*

*comparing the m6A-seq to the input (RNA-seq) of the same sample. 3) We don't put*

*much emphasis on absolute numbers of m6A peaks, rather differences in m6A peaks*

*between samples. Yes, the 6-week samples have less sequencing reads, but they also*

*have less m6A peaks after normalizing for library size and taking into account RNA-seq*

*information. However, to address the reviewers concerns we performed m6A*

*quantification with LC-MS/MS. We found that m6A levels are initially high then*

*decrease by 6 weeks, then steadily increase again reaching the highest level at 52-*

*weeks.*

*We also prepared PCA plots that show a high degree of similarity between replicates*

*and differences between tissue type (Supplementary figure S2).*

*Studies have shown that MACS2 is a suitable tool to analyze MeRIP-seq data. For*

*example, work by Liu et al., 2014 compared MACS2 and exomePeak and showed a*

*significant correlation between the results obtained on MeRIP-seq datasets using the*

*two techniques. And a more recent study by McIntyre et al., 2020 drew the same*

*conclusions, stating that most people use MACS2 to analyze MeRIP-seq and they*

*found it to be a reliable tool. In fact, these authors found MACS2 outperforms exomePeak. Another study by Anatanaviciute et al., 2017 also shows that MACS2 was*

*a better performer than exomePeak. Since we use the same parameters of MACS2 to*

*call peaks in every context we can call differential peaks. Lastly, we do not find any*

*literature mentioning that MACS2 is not accurate at calling differential peaks. In any*

*case we always inspect peaks and differential peaks visually using the IGV genome*

*browser and screenshots are provided as evidence in the figures. We have cited these*

*works in the Methods section to highlight our motivation for using MACS2.*

3)Throughout the manuscript, the authors should address and clarify 'm6A specificity'

vs. 'gene expression specificity' (e.g. Figures 1D, Fig 2, etc.). My impression is that

most of the 'differential m6A' described here is simply due to the relative expression of

the host mRNA rather than reflecting dynamic m6A modification. Accordingly, many of

the enriched GO pathways could simply from the tissue/age specific gene expression.

*We thank the reviewer for this comment and refer to the response given above for*

*point 2. In short, we are able to highlight differentially methylated transcripts because*

*we control for library size and differences in mRNA levels between samples.*

4)PCA analysis should be performed to check the m6A distribution among different

tissues and among brain development time points. Meanwhile, reproducibility should

be checked using correlation analyses between replicates.

*We have included PCA plots that show a high degree of similarity between replicates*

*and differences between tissue type.*

5)There is no experimental validation to support the authors' conclusions. More

stringent validations, including differential translation validation using western blots,

and expression of reporters with 3' untranslated region (UTR) are definitely needed.

*In the revised manuscript, we have included the data of Western blot and qRT-PCR on*

*selective genes validating what we observed bioinformatically regarding the differential*

*m6A methylation in AD.*

6)m6A has been reported in regulating many post-transcriptional regulation processes,

such as translation, alternative polyadenylation (APA), and alternative splicing. Splicing

should be also examined and included in the results.

*We thank the reviewer for this comment, we have taken this suggestion on board and*

*performed deep RNA sequencing to evaluate effects of m6A on splicing throughout*

*neurodevelopment. We find that m6A sites are associated with exon inclusion events*

*consistently across neurodevelopment. Please see the last paragraph under the*

*section Temporal dynamics of m6A methylation during early neurodevelopment, and*

*supplementary table S4 and supplementary figure S3.*

7)The authors should use more specific sequencing technology to evaluate the APA

events, such as PAS-seq, PolyA-seq, and PAT-seq.

*Whilst we agree with the reviewer that there are specific sequencing techniques to*

*evaluate APA events, the approach we took was to use a specific software that*

*evaluates APA usage from poly(A) isolated mRNA-seq. This provides us with the same*

*information that we would gather from PAS or PAT-seq.*

8)Page 6, line 33-34, the authors cannot use gene expression changes to indicate

mRNA stability. The expression difference can be due to both transcription differences

and/or RNA degradation. Besides, it is not accurate to use the gene expression to

demonstrate that the m6A changes in AD resulted in RNA turnover. Transcriptional

inhibition with Actinomycin D treatment should be used to study mRNA stability (or halflife).

*We understand the reviewer’s comment here and will take care to refer to mRNA*

*expression changes and not mRNA stability in the manuscript. It would not be feasible*

*to carry out actinomycin D treatments on the brain tissues that we used. Rather we*

*have amended the text to show that our analyses suggest there is a correlation*

*between m6A and mRNA expression.*

9)A comparative analysis of m6A genes between mouse and human should be

performed to study whether the m6A genes show the same trend between mouse and

human brains.

*A comparative analysis between mouse and humans was performed. Figure 3 shows*

*this. We compare m6A sites detected in mouse compared to human for all called m6A*

*sites in adolescent, old and differential m6A sites. We also include a gene ontology for*

*mouse and human which shows many similar biological processes between the two*

*species.*

10)For the Ribo-seq data, the authors used the publicly available Ribo-seq data of

YTHDF1 and YTHDF2 knockdown samples (in HeLa cells), which is not directly

relevant to their story. Ribo-seq sequencing on the AD disease model or iPSCs from

AD disease sample should be conducted to check the differential translations.

Moreover, the data analysis and conclusions from the YTHDF1 paper (Wang et al.,

Cell 2015; citation #5 in the current manuscript) have been recently called into question

(Zaccara and Jaffrey, Cell 2020), and therefore not considered satisfactory for this

work.

*We thank the reviewer for this comment. The paper published by Zaccara and Jaffrey,*

*Cell 2020 was not yet available when this manuscript was submitted and does bring*

*into question the Ribo-seq dataset by Wang et al., 2015 that we used in this study. The*

*Ribo-seq data was included only to support the main proteomic data which highlighted*

*the decrease in protein levels of differentially m6A methylated transcripts in 5XFAD*

*compared to WT. That is the main point of Figure 5. Therefore, after consulting with the*

*Editor, we have decided to remove the panel making use of the Ribo-Seq data (Figure*

*5E).*

11)The authors should pay more attention to m6A modifications of the AD-related

genes (https://www.alzforum.org/genetics). Whether these genes show different m6A

modifications, mRNA expression, and/or translation in AD models?

*We thank the reviewer for this suggestion. We observe a decrease and increase in*

*METTL3 and FTO in our RNA-seq data and in the publicly available proteomic data*

*that we used in the study. This observation has been added to the text.*

12)Figure 5A: The conclusion of global reduction simply based on sequencing data is

problematic and unconvincing. Again, the authors should try to use spike-in RNA

and/or mass spec to validate this possible change.

*The plot is a metaplot distribution of m6A over the gene based on normalized reads.*

*However to make the plot more convincing, we focused on the normalized read density*

*around called 3’ UTR m6A peaks in WT and 5XFAD and have also included the input*

*RNA-seq to absolutely show that the change in the profiles is due to differences in m6A*

*levels, and not due to mRNA expression.*

13)Figure 5D, and page 10, line 55, 'suggesting that m6A is not a major regulator of

mRNA stability in these transcripts'. The authors should explain the discrepancy with

their conclusion in Figure 1, where they found that m6A is related to the

expression/stability of the host mRNA. Since AD is a age-related phenotype, the effect

should be consistent.

*We thank the reviewer for this comment. The subset of genes that we found to be*

*differentially methylated in 5XFAD compared to WT were AD-specific rather than agespecific as evidenced by the gene ontology analysis. In this particular subset of*

*differentially methylated transcripts we find that m6A correlates with protein levels*

*rather than mRNA levels. Furthermore, we don’t claim that m6A only regulates mRNA*

*levels during neurodevelopment. For example, we also now show that some m6A*

*coding sites are associated with exon inclusion events. Of course, m6A has many roles*

*and these are just two that we observe in a subset of m6A-marked transcripts during*

*neurodevelopment.*

14)The authors should explain and justify why they chose fly for validation instead of mice here?

*Using the fly model allows us to easily determine the effects of m6A players on AD in a*

*facile, fast, qualitative manner compared to mice. We employed the GMR-GAL4 driver,*

*which expresses the yeast transcription factor GAL4 under the control of glass multiple*

*reporter (GMR) promoter elements, to express the Tau gene specifically in the eye.*

*Thus, we can determine whether m6A players have a role in AD by easily observing*

*changes in eye phenotype. Using the Drosophila system is a much more rapid and*

*visual representation of the effect on m6A players in AD, which is not easily possible*

*with mice. We have added text to make this clearer.*

15)The experimental design in the fly validation is very confusing. In figure 5, the

authors found that the YTHDF1 could be the driver of the m6A-mediated phenotype,

but why did they choose YTHDF2 instead in the RNAi experiment in fly?

*We thank the reviewer for this comment. There is in fact only one ortholog for the*

*YTHDF proteins in Drosophila. We will amend the figure, legend and main text to make*

*this clearer.*

Other minor points:

1.Figure 1B: This distribution plot looks weird. What do the START and STOP here

mean? If it's start codon and stop codon, then why the 3'UTR are largely depleted

while Fig 1A shows there are quite a lot 3'UTR peaks?

*We ended up changing Figure 1 and decided to remove this panel. Instead we*

*included the m6A quantification by LC-MS/MS. But to answer the question, yes, start*

*and stop relates to the start and stop codon. The pie charts (which have now been*

*removed for bar graphs) show absolute numbers of m6A peaks, whilst the distribution*

*plot shows normalized read counts. After taking into account gene region length, this*

*plot actually shows an enrichment of m6A around the stop and 3’UTR as previously*

*noted in the literature. For example, there are more m6A peaks in the CDS than any*

*other region, but after accounting for the longer size of the CDS compared to UTRs,*

*there is no enrichment of m6A found in the CDS.*

2.Figure 1C: I couldn't figure out from which peak set the authors got this motif?

Perhaps the authors can compare the motif enrichments in different dataset to see if

there is peak quality difference between samples.

*The motif was generated using 2-week old cortex data. The legend will be amended to*

*note this. All samples, though, were found to harbor the GGAC motif that has been*

*previously reported.*

3.Figure 5E: Why didn't the authors directly compare the translation efficiency between

AD vs non-AD here? Which would be much more straightforward.

*This panel has been removed as discussed above.*

**Second round of review**

**Reviewer 1**

The authors have addressed all of my concerns.

**Reviewer 2**

Overall the authors have adequately addressed most concerns and where necessary included additional data. I am still not convinced by many of their peak calling based m6A differential analyses, but they added the Mass spec result (Fig 1B in revised manuscript), which makes it more convincing. Nevertheless, in their response to previous comment #2: I don't completely agree with the author's argument. I understand that MACS2 would adjust for sequencing depth and normalize to input sample, but the sequencing depth can still affect the peak calling sensitivity. Let's say you have a meRIP-seq with 20M reads, if you downsample it to 15M, it is very likely that you will get fewer peaks. That's why I think it's not appropriate to use peak number to represent the global m6A level in the original manuscript. But I appreciate the mass spec results in the revised manuscript, which makes it generally more convincing.
